# Bacteria enable tolerance to bile salt exposure in an immune-competent human intestinal model

**DOI:** 10.1080/19490976.2025.2514144

**Published:** 2025-06-21

**Authors:** Yuan Li, Chia-Ming Wang, Peng Zhao, Matthew T. Fernez, Rebecca L. Carrier

**Affiliations:** a Chemical Engineering Department, Northeastern University, Boston, MA, USA; b Bioengineering Department, Northeastern University, Boston, MA, USA

**Keywords:** *In vitro* intestinal model, bile salts, commensal microbes, permeability, viability, crosstalk

## Abstract

Bile salts (BS) are known to be highly important in modulating bacteria-host crosstalk in the gut, serving as essential signaling molecules governing intestinal homeostasis. However, understanding of this crosstalk is limited by challenges of analyzing the mucosal interface *in vivo* and a lack of *in vitro* intestinal models incorporating bile, bacteria, and host cells. In this study, the impact of bile micelles on an *in vitro* intestinal model integrating commensal microbes with immune-competent human duodenal epithelium was studied. Physiological concentrations of BS/phosphatidylcholine (PC) micelles comprising a model bile were damaging to epithelial cells under hypoxic but not normoxic conditions. However, incorporation of a commensal bacterial consortium protected the epithelium from bile micelle-associated damage, as reflected in increased epithelial cell viability and preserved monolayer barrier function. Furthermore, the model bile enabled homeostasis when bacterial consortia were incorporated into epithelial-immune co-cultures, reducing barrier damage and inflammatory response induced by the consortia and modulating bacterial growth. In considering factors lacking *in vitro* that may promote homeostasis when intestinal tissue is exposed to bile and bacteria, we investigated the influence of mucus on bacteria-bile-epithelial/immune crosstalk by adjusting mucus thickness using air–liquid interface culture. Thicker mucus layers not only impacted the growth of consortium strains, resulting in enhanced growth of the mucin metabolizer *Bifidobacterium longum*, but also reduced inflammatory responses to bacteria and bile-induced epithelial damage. Overall, this study introduces a human immune-competent intestinal model capturing key features of bacteria-bile-mucosal crosstalk. Studies conducted with this system demonstrated the importance of oxygen levels, commensal microbes, and the mucus barrier in maintaining homeostasis in the small intestinal mucosa in the context of bile micelle exposure.

## Introduction

Bile acids (BA) are present at high concentrations in the upper gastrointestinal tract, predominantly in their conjugated salt form,^1^ yet are not included in most *in vitro* intestinal models. Originally recognized for their roles in facilitating the digestion and metabolism of lipid products, they are now understood as a family of hormones and antimicrobial molecules that regulate intestinal mucosa homeostasis.^2–5^ In humans, these compounds are produced in the liver via conversion of cholesterol to BS in conjugated primary form. These BS are stored in the gallbladder and postprandially secreted into the duodenum, contributing to the digestion of lipids and other nutrients.^6^ The majority of BS are reabsorbed in the distal small intestine.^7^ During their journey in the intestine, a portion of BSs are converted by commensal bacteria to unconjugated primary BAs through bile salt hydrolase (BSH) and subsequently further converted to secondary BS.^7^

BS have important signaling functions through interactions with cell receptors including Farnesoid X receptor (FXR), Takeda G protein-coupled receptor 5 (TgR5), and Vitamin D receptor (VDR).^8^ Binding to these receptors and subsequent signaling contributes not only to antimicrobial function but also other essential regulatory roles, for example, regulation of cell proliferation and host metabolism.^9–11^ Conjugated cholic acid (CA) and chenodeoxycholic acid (CDCA) are particularly abundant in the human intestine, comprising approximately 80% in total of the duodenal bile pool.^12^ Studies have shown that conjugated cholic acid exhibits anti-inflammatory effects in mouse models of ulcerative colitis, suggesting promise as a potential inflammatory bowel disease (IBD) treatment.^13^ Taurine-conjugates (TCA) have been demonstrated to inhibit host cell damage caused by *Clostridium difficile* toxins^14^ and exhibit antimicrobial functions at high concentration (>100 mM).^15^ CDCA and its glycine-conjugate BS GCDC have high binding affinity for FXR, a receptor that regulates mucosal immune homeostasis^16,17^ by inducing the expression of genes that produce factors to prevent bacterial overgrowth and promote epithelial integrity.^18^ While present at lower concentrations in the upper GI tract, secondary BS also have important functions. For example, the hydrophilic BS tauroursodeoxycholate (TUDC) has been reported to alleviate intestinal inflammation and barrier disruption in mice, inhibiting experimental colitis by preventing early intestinal epithelial cell death and suppressing cell apoptosis induced by TNF-α and LPS.^19^

Alongside their positive effects, reports also suggest that BA can disrupt epithelial homeostasis by increasing cell permeability and inducing intracellular stress.^8,20^ Interestingly, *in vitro* studies indicate that while unconjugated BA can cause intestinal epithelial monolayer delamination, the corresponding conjugated primary BS can alleviate these negative effects at physiologically relevant concentrations.^21^ Inhibition of bile salt deconjugation *in vivo* using a small molecule inhibitor further supported protective action of conjugated BS against increased permeability and inflammation.^21^ Cholesterol and phosphatidylcholine also contribute to protection of host cells from bile salt-induced injury via the formation of micelles with BS.^22,23^

Thus, the impact of bile salt exposure on intestinal function is complex and depends on various interrelated factors. Current experimental models used to study the relationships between BS and the intestinal mucosa are limited in relevance: xenogeneic animal models do not recapitulate the human response, and *in vitro* models are based largely to date on cancerous cell lines. Mice are commonly used for *in vivo* studies, yet they exhibit a bile profile very different from that in humans, notably lacking glycine-conjugated BA in the intestine.^21,24^ In addition, humans are reported to predominantly synthesize CA and CDCA, whereas CA and β-muricholic acid are the predominant products in mice.^25^ Cell lines commonly used to model intestinal epithelium *in vitro*, such as Caco-2 and T84 cells, are not only carcinoma cell models but also originate from large intestine tissue, and thus may not closely represent cell response in the bile-rich small intestine.^26–28^ Intestinal organoids, while highly useful tools for studying intestinal physiology,^29^ present challenges associated with apical exposure and permeability assessment, as differentiated organoids generally have apical cell surfaces facing an enclosed lumen compartment.^10^ Perhaps most importantly, most *in vitro* systems lack key features of the intestinal environment that mediate interactions between BS and intestinal cells, including bacteria. For example, specific commensal bacteria are known to have bile salt hydrolase, dehydroxylation, and hydroxysteroid dehydrogenase activity and thus regulate the composition of the BA pool with significant impact on intestinal development and health.^30,31^ Due in part to the antimicrobial function of BS, the profile of BS in turn influences the composition of gut microbiota.^32^ Further, BA modulate function of innate immune cells in the intestine such as dendritic cells (DCs), which are crucial to host reactivity to gut microbiota and maintenance of tolerogenic phenotype.^33^

Investigation of the important and complex relationships between commensal microbes, BS, and host cells (including epithelial cells and immune cells) at the intestinal mucosal interface *in vitro* presents notable challenges. These challenges include stabilizing bacteria density in co-cultures, design of medium composition to support multiple bacterial strains and host cells during co-culture, potential toxicity of BAs to host cells and bacteria, and the necessity of a low oxygen environment for some gut bacteria. Indeed, the exact role of luminal oxygen levels in BA-host cell interactions remains undetermined yet reported impact of hypoxia on commensal bacteria, and host cell activities^34,35^ suggest it may be an important factor.

In this study, we established a primary human small intestinal *in vitro* model capturing key physiological features for analyzing bile-bacteria-host cell crosstalk. The model is comprised of a simplified BS pool including phosphatidylcholine for micellar solubilization, human organoid-derived duodenal epithelial monolayers, dendritic cells pivotal for innate inflammatory responses to luminal cues, and a simplified microbial consortium consisting of commensal strains reported as abundant in the human intestine^36^ or representative of probiotic and lipid metabolism-related functions.^37,38^ We utilized this immune-competent intestinal model to demonstrate that inclusion of the microbial consortium mitigates damage induced by bile exposure under hypoxia. In turn, the model bile modulated the consortium composition and mitigated immune response and loss of viability and barrier function induced by incorporation of the bacterial consortium. Furthermore, we tested if the system could capture characteristic features of response to a high fat stimulus observed *in vivo*.^37^

## Materials and methods

### Monolayer generation

Primary human duodenal epithelial stem cells used in this study were derived from duodenal organoids (donor H416) obtained from the Harvard Digestive Diseases Center (HDDC) Organoid Core at Boston Children’s Hospital. Duodenal crypt tissues were obtained from tissue biopsies (grossly normal appearing regions of the duodenum). Duodenal organoids were expanded in Matrigel® matrix (Corning 354,234) with expansion medium (EM) and passaged every 7 d. EM was prepared using 50% v/v Wnt, R-spondin, noggin (L-WRN) conditioned medium (Breault Lab HDDC Organoid Core) with 40% v/v Advanced Dulbecco’s Modified Eagle Medium/F-12 (Advanced DMEM/F12, Gibco™, 12634010) supplemented with 1X GlutaMax (Thermo Scientific 35,050,061), 10 mM HEPES (Thermo Scientific 15,630,080), 10 nM gastrin (Sigma Aldrich, G9145), 1× N2 (Thermo Scientific 17,502,048), 1× B27 (Thermo Scientific 12,587,010), 50 ng/mL murine EGF (PeproTech, 315–09), 1 mM N-acetyl-cysteine (Sigma Aldrich, 616–91–1), 10 mM nicotinamide (Sigma Aldrich 240,206), 10 μM Y27632 (TOCRIS, 1254), 5 nM A83–01 (TOCRIS, 2939), 3.3 μM SB202190 (TOCRIS, 1264) and 50 μg/mL Primocin (InvivoGen, PML-42–07). To culture the duodenal epithelial monolayer, 300 μL of 150 μg/mL rat tail collagen I (Corning 354,236) in 1X phosphate buffer saline without calcium and magnesium (PBS, Gibco 14,190–144) were added to each apical chamber of a 24-well Transwell® insert system and incubated for 1 h. After 1 h coating, inserts were rinsed once using EM. Duodenal organoids (passage 15–20) were harvested on day 6 or 7 of culture by physically scraping organoids embedded in Matrigel® matrix (25 μL of Matrigel® for each droplet) off the tissue culture plate, followed by centrifugation at 300 g for 5 min at room temperature (RT). After the centrifugation, supernatant was discarded, and the organoid pellet was resuspended with 3 mL of 0.5 mM ethylenediaminetetraacetic acid disodium salt (EDTA, Sigma-Aldrich, E6635) in 1X PBS to dissolve Matrigel®. After centrifugation at 400 g for 5 min at RT, the supernatant was discarded. The organoid pellet was then mixed in 1 mL of trypsin (Gibco 25,200,072) for 5 min at 37°C for organoid dissociation. The resulting mixture of organoids and cells was further dissociated into single cells by manually pipetting with a 1000 μL bent tip. Trypsin was neutralized with 5 mL of Advanced DMEM (Gibco 12,491,015) supplemented with 10% fetal bovine serum (FBS, R&D Systems, S11150), 1X GlutaMAX, and 1% penicillin–streptomycin (Gibco 15,140,122), followed by centrifugation at 400 g for 5 min at RT to obtain the cell pellet. 2.5 × 10^5^ single cells were seeded on collagen-coated 24-well inserts and cultured 4–5 d with EM until they formed a confluent monolayer. The culture medium was then switched to differentiation medium (DM) (Table S1). Autoclavable 000 size rubber stoppers (Cole Parmer, EW-62990–00) were precut and sterilized to fit the Transwell® inserts. Two days after differentiation was initiated, anaerobic co-culture was established by sealing inserts’ apical chamber with rubber stoppers and switching apical medium to the deoxygenated 10% M9 modified medium (Table S2) in 1X Dulbecco’s phosphate buffer saline with calcium and magnesium (PBS (+/+), Gibco 14,040–133). For air–liquid interface (ALI) culture, DM was only used in the basolateral compartment and supplemented with 300 ng/mL vasoactive intestinal peptide (VIP, ANASPEC, AS-22872), 1.4 nM prostaglandin E2 (PGE2, Prepotech 3,632,464), and 10 μM gamma-secretase inhibitor (DAPT, Sigma-Aldrich, SCP0004). The apical compartment was left empty after differentiation was initiated. VIP is an endogenous intestinal hormone which mediates the luminal water homeostasis, supporting a hydrated mucus layer of uniform thickness.^39^

### Generation of DCs via monocyte differentiation

Human DCs were differentiated from human peripheral blood monocytes (PBMCs, STEMCELL Technologies, isolated from whole blood according to supplier protocols) for 7 d in dendritic cell differentiation medium: Advanced Roswell Park Memorial Institute 1640 (Advanced RPMI 1640, Gibco 12,633,012) supplemented with 1X GlutaMax, 1% penicillin-streptomycin, 10 nM retinoic acid (Sigma-Aldrich, 302–79–4), 100 ng/mL human GM-CSF recombinant protein (Gibco, PHC2015), 70 ng/mL recombinant human IL-4 Protein (SinoBiological 11,846-HNAE), 10% heat-inactivated fetal bovine serum (R&D Systems, S11550H), 1% MEM non-essential amino acid solution (Gibco 11,140,050). Seven days after initiation of PBMC differentiation and 4 d after commencement of monolayer differentiation, DCs were harvested and seeded on the basolateral membrane of the Transwell® inserts, as previously described.^40^ After seeding, the basolateral compartment was filled with 700 μL of antibiotic free DM for 24 h prior to consortium co-culture and BS/PC exposure.

### Exposure of human duodenal epithelial monolayer to model bile

Four days after commencement of monolayer differentiation, apical medium was changed to 10% M9 in PBS (+/+) containing model bile micelles, comprised of sodium taurocholate (NaTC, Sigma-Aldrich 86,339), sodium glycochenodeoxycholate (NaGCDC, Sigma-Aldrich 16,564–43–5), sodium tauroursodeoxycholate (NaTUDC, EMD Millipore 580,549) and phosphatidylcholine (PC, Avanti, 131601P). Three formulations of model bile micelles were prepared: Fasted State (FasS), Low Fed State (FedS-Low), Fed State (FedS) (Table 1). The types of BS and ratio to PC were selected based on previous reports of duodenal luminal fluid characterization, where conjugated cholic acids and chenodeoxycholic acids are the most abundant BA, primarily conjugated with glycine and taurine.^41,42^ Indeed, cholic acid and chenodeoxycholic acid are the two primary bile acids produced in the human liver and together comprise over 70% of the bile acids within human duodenal fluid.^41^ NaTUDC is present at lower levels in the human intestine but has been reported to offer beneficial effects to the host. The chemically defined M9 modified medium (Table S2) was determined to support the simultaneous growth of the four commensal strains included in the bacterial consortium used in this study (Figure S1). The total BS concentrations used matched physiological concentrations of 4 mM (Fasted State) and 12 mM (Fed State) in human duodenal luminal fluid.^41^ To obtain BS/PC micelles, 0.1 mL of 100 mM PC in chloroform was evaporated in a vacuum chamber overnight to generate a PC film. The PC film was then reconstituted with 3.33 mL of 10% M9 modified medium in PBS (+/+) containing 10 mM HEPES and 12 mM BS. The FasS solution was obtained by further diluting the FedS solution three times using 10% M9 modified medium with 10 mM HEPES. Cultures were exposed to bile salt preparations for 24 h. Antibiotic free DM was maintained in the basolateral compartments.

**Table 1.** BS/PC concentrations in media.

Concentration (mM)	NaTC	NaGCDC	NaTUDC	PC
Fasted State model bile (FasS)	1.95	1.95	0.1	1
Low Fed State model bile (FedS-Low)	3.90	3.90	0.2	2
Fed State model bile (FedS)	5.85	5.85	0.3	3

### Co-culture with model bile exposure

Four bacterial strains were selected based on their relevance in the duodenum and/or their role in gut metabolism, and to capture some degree of phylogenetic diversity. Specifically, *S. mitis* (Phylum: *Bacillota*) and *P. melaninogenica* (Phylum: *Bacteroidotas*) were observed to be abundant components of the duodenal mucosa-associated microbiota,^36^ *B. longum* (Phylum: *Actinomycetota*) has been reported in the human duodenum and plays an important role in host health,^43,44^ and *C. bifermentans* (Phylum: *Bacillota*) is associated with host lipid metabolism.^37^ Forty-eight hours before bacterial seeding, *B. longum KLE 2100* (gift from Kim Lewis Lab) and *P. menlaninogenica ATCC 25845* were revived and expanded from −80°C using Yeast Casitone Fatty Acids with Carbohydrates Broth Medium (YCFAC, Anaerobe System). Twenty-four hours before seeding, *C. bifermentans ATCC 638* and *S. mitis NCIMB 13770* were revived and expanded in Nutrient Broth No.1 (Sigma-Aldrich 70122) and Reinforced Clostridia Medium (RCM, Millipore 105411), respectively. To prevent oxygen exposure prior to initiation of intestinal model bile salt exposure, bacterial suspensions were prepared in an anaerobic chamber using 10% M9 modified medium with 10 mM HEPES supplemented with or without 4 mM/1 mM (FasS) or 12 mM/3 mM (FedS) BS/PC at 1.33 × 10^5^ units/mL. Bacterial mixtures were collected by 1 mL syringe (BD®) with 28-gauge needle for seeding into Transwell® inserts at 4 × 10^4^ units/well (1 × 10^4^ units of each strain/well). After seeding, the stopper injection holes were sealed by petroleum jelly (Vaseline®). After 24 h of co-culture in the 5% CO_2_ incubator, apical medium was collected into pre-deoxygenated anaerobic culture tubes (Chemglass Life Sciences, CLS420801), DNA from dead bacteria was blocked with 8 μg/mL ethidium monoazide bromide (EMA, Biotium 40015), and the live bacterial DNA from spent medium was extracted for qPCR analysis as described in section 2.8. Another 150 μL of basolateral spent medium was stored at −20°C for enzyme-linked immunosorbent assay (ELISA) assay. For co-culture under high-fat stimulation, 50 μM oleic acid (OA, Thermo Scientific 31,997.14) was incorporated into 10% M9 modified medium with or without FasS solution. The OA solution, with or without FasS, was then combined with the consortium and injected into the apical compartment of the stopper-sealed ALI Transwell® system.

### Live/Dead assay

After BS/PC exposure and/or bacterial co-culture, intestinal epithelium viability was assessed. Twenty-four hours after BS/PC and/or bacteria introduction, the rubber stopper was removed, and apical spent media was collected. Three hundred microliter of Live/Dead staining assay solution (Invitrogen, L3224) consisting of 2.0 μM Calcein AM (staining live cells green) and 4.0 μM ethidium homodimer-1 (staining dead cells red) in PBS (+/+) was added to the apical chamber and incubated for 30 min at 37°C. After incubation, both apical staining solution and basal medium were discarded. Membranes were rinsed on both sides three times by PBS (+/+) and imaged using an Olympics IX51 fluorescence microscope.

### Imaging of F-actin, Ki67, ZO-1, and mucus

At the end of the bile salt exposure and co-culture experiments, epithelial monolayers were fixed with 4% paraformaldehyde (Thermo Fisher Scientific 28908) for 10 min at RT. They were then incubated with 0.1% Triton-X 100 (Sigma, T8787) for 5 min at RT and rinsed three times with PBS, followed by treatment with blocking reagent (Santa Cruz, SC-516214) for 30 min at RT. To label F-actin, cells were stained with Alexa Fluor 633-conjugated phalloidin (Invitrogen, A22284) at a 1:400 dilution in blocking reagent for 90 min at RT while protected from light and rinsed with PBS three times. To label Ki67, followed the F-actin staining, cells were stained with 1:100 blocking reagent diluted Alexa Fluor 488-conjugated Ki-67 antibody (Santa Cruz, SC-23900) for 90 min at RT and rinsed with PBS three times. To label zonula occludens-1 (ZO-1) tight junction protein, cells were stained with Alexa Fluor 488-conjugared ZO-1 antibody (Invitrogen, MA3–39100-A488) at a concentration of 5 µg/mL in blocking reagent for 90 min at RT while protected from light and rinsed with PBS three times. After F-actin or ZO-1 staining, cells were stained with 4′,6-diamidino-2-phenylindole (DAPI, Invitrogen 62248) at 300 nM concentration for 15 min at RT, then rinsed with PBS three times. For mucus staining, the monolayer was fixed and stained with 5.0 µg/mL Alexa Fluor 647 conjugated Wheat Germ Agglutinin (WGA, Invitrogen W32466) for 30 min at RT. Subsequently, nuclei were stained DAPI from basolateral side for 20 min. After staining, the apical side was gently rinsed twice, and the basolateral side was rinsed three times with PBS. Samples were imaged using a Zeiss LSM 800 confocal microscope.

### ELISA assay for dendritic cell inflammatory response

Fifty microliter of medium from the basal compartment was collected pre-/post bile salt incubation and used for analysis of concentrations of TNF-α and IL-10 via ELISA. Before TNF-α measurement, basolateral medium was diluted three times to bring the concentration within the assay range. Human IL-10 ELISA kit (Thermo Fisher Scientific, EHIL10) and Human TNF-α ELISA kit (Thermo Fisher Scientific, KHC3011) were used for ELISA analysis per manufacturer’s instructions.

### qPCR and RT-qPCR analysis for quantification of epithelial cell gene expression and bacterial composition

Twenty-four hours after commencement of bacterial co-culture, apical spent media were collected in the pre-deoxygenated Chemglass anaerobic culture tubes and treated with 10 μM EMA under light for 20 min. After EMA treatment, spent medium was centrifuged at 13,000 g for 3 min. Supernatant was discarded, and 100 μL cold deoxygenated PBS was used to resuspend the pellet. The microbial genomic DNA was extracted from the EMA-treated sample by Monarch gDNA Purification Kit (New England Biolab, T3010S) according to manufacturer’s instructions. The density of each strain and consortium composition were quantified by qPCR using Luna® Universal qPCR Master Mix (New England BioLab, M3003) according to manufacturer’s instructions with primers listed in Table S3.

For epithelial cell gene expression, after 24 h exposure to BS/PC with/without OA and bacterial consortia, cells on the culture insert membrane’s apical side were lysed, and the mRNA from cells was extracted and purified using RNeasy Micro Kit (QIAgen 74004) according to vendor’s instructions. The cDNAs were synthesized using QuantiTect Reverse Transcription Kit (QIAgen 205311) following manufacturer’s instructions. All cDNA samples were stored at −80°C for RT-qPCR analysis. Gene expression levels were determined using the ΔΔCT method normalizing target gene to glyceraldehyde 3-phosphate dehydrogenase (GAPDH) housekeeping gene. Six types of TaqMan gene expression assays (Table S4): GAPDH, phosphoinositide-dependent kinase-1 (PDK1), yes-associated protein 1 (YAP1), nuclear factor erythroid 2-related factor 2 (NrF2), diacylglycerol acyltransferase-1 (DGAT1), and diacylglycerol acyltransferase-2 (DGAT2) were used according to manufacturer’s instructions.

### Permeability assay

To quantitatively test the integrity of the epithelial monolayer, permeability was measured using Lucifer Yellow (LY) assay. Briefly, the spent medium in the apical compartment was removed, and PBS (+/+) was added to gently rinse. LY solution was prepared from Lucifer Yellow CH dilithium salt (Sigma-Aldrich, L0259-25 MG) at 100 µM concentration in DM. Three hundred microliter of LY solution was added to the apical compartment and 700 μL of DM to the basolateral compartment, followed by incubation at 37°C for 60 min. After incubation, the medium in the basolateral chamber was collected and analyzed in a BioTek® SYNERGY H1 microplate reader at an excitation of 485 nm and an emission of 535 nm. The fluorescence values of a set of standards were measured to create a standard curve. The permeability of the monolayer was calculated using the following equation:

$$P_{\mathrm{app}}=\frac{V_{\mathrm{rec}}\cdot C_{\mathrm{rec}}}{A\cdot t\cdot C_{\mathrm{don}}}$$

where V_rec_ is the volume of the receiver (basolateral) compartment, C_rec_ is concentration in the receiver compartment, A is the seeded area, t is incubation time, and C_don_ is the concentration in the donor (apical) compartment.

### BS/PC medium pH measurement and micelle size characterization

To measure the pH values of the apical co-culture medium, 2 mL of 10% M9 modified medium with or without BS/PC micelles and with or without OA were deoxygenated in an anaerobic chamber for 12 h. Following the deoxygenation, pH of the medium was measured using a pH meter. To measure micelle size, 10% M9 modified apical medium with 1 mM of PC, and 10% M9 modified apical medium with 4 mM/1 mM BS/PC were prepared as described in section 2.3. One milliliter of each solution was transferred to a cuvette and analyzed in an Anton Paar particle size analyzer. The micelles’ hydrodynamic sizes were measured at RT, and the particle size characterization was displayed as number distribution.

### Statistical analysis

All cell experiments were conducted with at least three independent biological replicates (*N* ≥ 3), and the images presented in the results section are representative examples. Statistical analyses were performed using GraphPad Prism, with significance assessed via one-way analysis of variance (ANOVA), and unpaired t-test. All quantitative data are presented as mean values with standard error of the mean (SEM), with each dot representing one replicate. Statistical significance was determined using ANOVA Tukey’s multiple comparison test or unpaired two-tailed t-test with a threshold of: *p < 0.05, **p < 0.01, ***p < 0.001, ****p < 0.0001.

## Results

**Figure 1.**
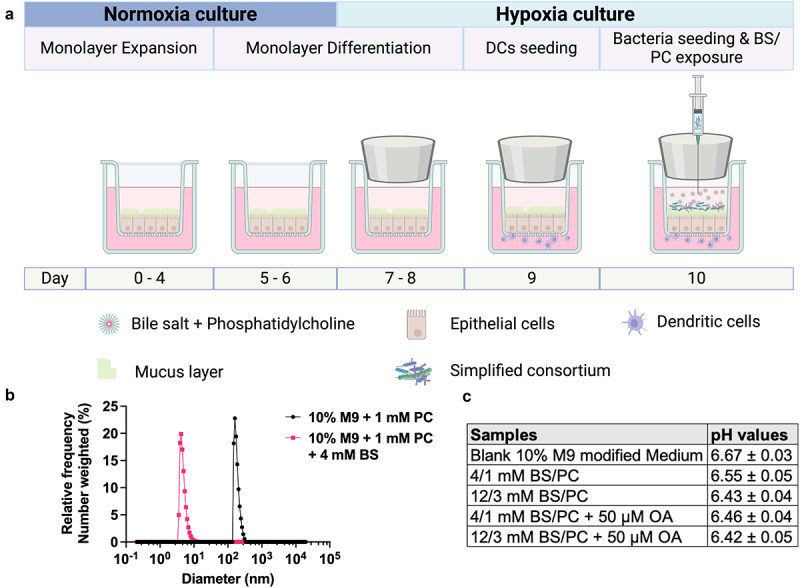
Co-culture of immune-competent human duodenal epithelium with obligate anaerobic gut bacterial consortium and bile exposure. (a) Schematic diagram of the 3D co-culture system including intestinal epithelial monolayer, dendritic cells, and anaerobic gastrointestinal microbiota using Transwell® inserts and rubber stoppers. (b) BS/PC micelles size distribution in the apical co-culture medium. (c) pH of defined apical media, including model bile.

Despite the significance of bile in modulating host-bacteria crosstalk and bacteria being recognized as crucial in regulating response to bile components, most host-bacteria *in vitro* models do not include bile components, and most *in vitro* bile–cell interaction studies to date have not considered the impact of bacteria. Further, *in vitro* host-bacteria and bile–cell interaction studies generally do not consider the impact of physiological hypoxia. To investigate microbe-bile-host cell interactions, we limited oxygen exposure to the apical compartment of culture inserts using rubber stoppers (Figure 1(a)). This system configuration was previously shown to create a <0.5 mg/L O_2_ or <10 Torr pO_2_ hypoxic environment supporting anaerobe culture.^45,46^ It includes two compartments separated by an epithelial monolayer and the porous membrane of the insert, where the upper part represents the hypoxic lumen containing bile components and bacteria, while the lower part represents the normoxic stromal space with immune cells. This 3D model incorporates minimal but essential components central to intestinal bacteria-bile-epithelium/immune crosstalk.

### Primary human immune-competent intestinal model exhibits varying levels of tolerance to prolonged bile exposure under normoxia and hypoxia conditions

To first assess the ability of the primary epithelium-immune model to withstand exposure to bile components in the absence of bacteria, we introduced model bile (Table 1) to the apical compartment at concentrations representing those reported during the fasted (FasS) or fed (FedS) states in the human proximal intestinal lumen both under normoxia (without stopper in apical compartment) and hypoxia. The model bile included primary BS prominent in the small intestine, as well as a secondary bile salt (NaTUDC) demonstrated to be anti-inflammatory.^19^ The presence of model bile micelles in these media was confirmed by visual inspection, as large PC aggregates observed in the absence of BS were no longer present upon addition of BS, supporting the micellar solubilization of PC by bile salt molecules. Further, micelle particle size was measured and found to be 8–10 nm (Figure 1(b)), in the range of bile micelle sizes reported in the literature.^47^ The pH value of the apical formulations ranged from 6.4 to 6.7 (Figure 1(c)), which is in the range of the human duodenal luminal pH.^42^

**Figure 2.**
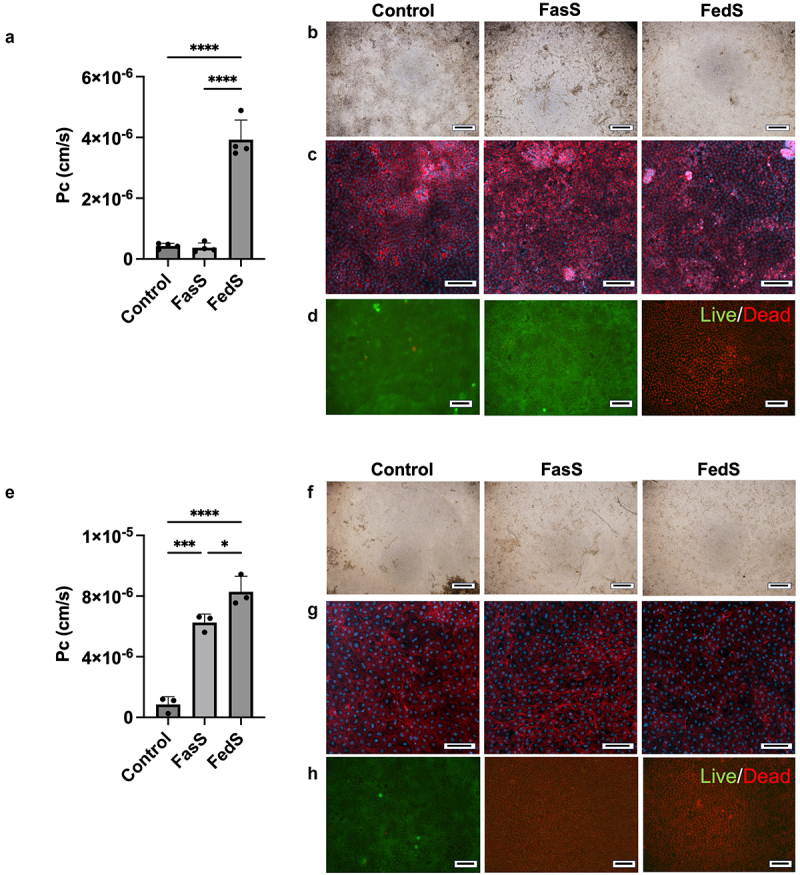
Intestinal model tolerance to BS/PC micelles is greater under normoxia than hypoxia. (a-d) Normoxia: impact of 24 h exposure to BS/PC at concentrations reflecting fasted or fed states on a) monolayer permeability (Pc), (b) structure as reflected by bright field imaging, (c) morphology as reflected by DAPI (blue) and F-actin (red) staining, and d) viability as reflected by live(green)/Dead(red) staining; (e-h): hypoxia: impact of 24 h exposure to BS/PC at concentrations reflecting fasted or fed states on e) monolayer permeability (Pc), (f) structure as reflected by bright field imaging, (g) morphology as reflected by DAPI (blue) and F-actin (red) staining, and (h) viability as reflected by live(green)/Dead(red) staining. (**Control**: No BS/PC). Scale bar: b, f 500 μm; c, d, g, and h 100 μm. Significant differences from one-way ANOVA analysis: *p < 0.05, ***p < 0.001, ****p < 0.0001.

FasS introduced to the apical chamber did not impact monolayer viability or barrier integrity after 24 h of culture in normoxia (Figure 2(a–d)). However, both viability and monolayer permeability were adversely affected, and the monolayer became more transparent after FedS exposure, despite no visible changes in structure. Compromised barrier function and disrupted tight junctions induced by FedS exposure were supported by observation of ZO-1 signal dispersing into the cytoplasm (Figure S2). These findings indicate that model bile was not toxic to the primary human intestinal model at a physiological fasted state concentration under normoxia. However, there is a tolerable concentration threshold for prolonged exposure of the monolayer to the bile complex. As fed state levels of bile are present in the intestinal lumen transiently after a meal, but not consistently over a prolonged period, this adverse effect of FedS over 24 h may reflect a physiological response.

Oxygen level is low (~5%) in the small intestinal lumen,^48^ and this low level of oxygen may regulate cell activities such as oxidation and metabolism.^34,35^ The 5% CO_2_ incubator air used to represent normoxia is thus an oxygen rich, non-physiologically relevant state for an intestinal model. Surprisingly, the FasS formulation that did not impact intestinal model permeability or viability during prolonged exposure in normoxia was toxic in hypoxia. While no visible changes in structure were observed (Figure 2(f,g)), cell death and elevated permeability of monolayers after 24 h of exposure were evident (Figure 2(e,h)). This suggests that low oxygen levels may decrease the tolerance of intestinal epithelial cells to bile. This result contrasts with reports of higher bile concentrations in human duodenal luminal fluid, approximately 4.3 mM for the fasted state and 12.6 mM for the fed state.^41^

### Commensal microbes protect epithelium from damage associated with FasS exposure and mitigate the toxicity of FedS model bile at low oxygen levels

**Figure 3.**
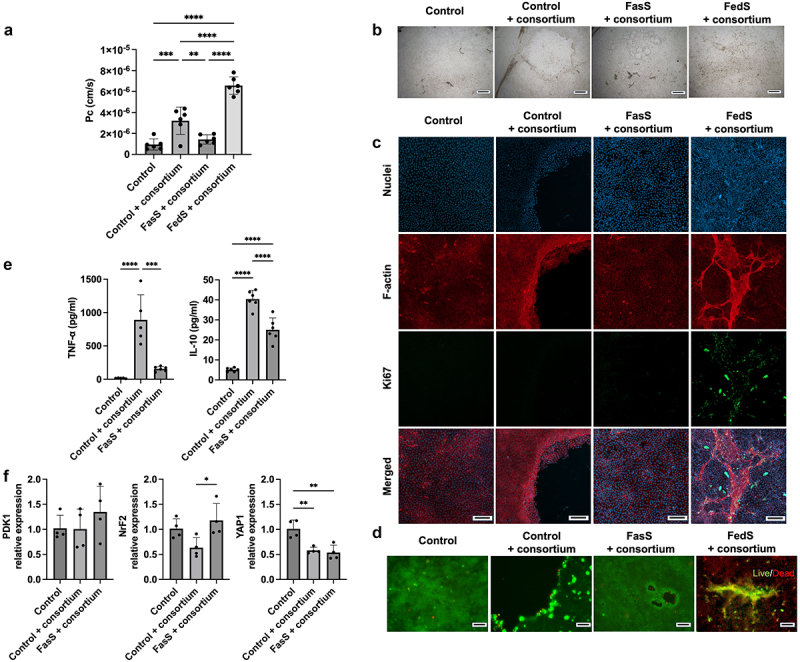
Bacteria increase intestinal model tolerance to BS/PC micelles, and BS/PC micelles mitigate barrier disruption, cell death, and immune responses to bacteria. Impact of 24 h exposure to BS/PC and/or bacteria under hypoxia on (a) monolayer permeability, structure as reflected by (b) bright field and (c) fluorescent staining (F-actin, red; Nuclei, blue; Ki67, green) (d) viability as reflected by live(green)/Dead(red) staining, e) immune response reflected in cytokine secretion, (f) oxidative stress-related gene expression. (**Control**: No BS/PC, no consortium). Scale bar: b. 500 μm, c&d. 100 μm. Significant differences from one-way ANOVA analysis: *p < 0.05, **p < 0.01, ***p < 0.001, ****p < 0.0001.

A simplified bacterial consortium was introduced to create bacteria-epithelium-dendritic cell co-cultures (BEDCC) as intestinal models. Incorporating bacteria into the intestinal model enabled maintenance of the epithelial cell barrier function, monolayer integrity, and viability during exposure to FasS under hypoxia (Figure 3(a–d), S3). These results suggest that the commensal microbes actively modulate bile-epithelial crosstalk, enhancing the bile tolerance of epithelial cells or reducing bile toxicity. However, exposure of BEDCC to FedS under hypoxia resulted in intact (Figure 3(b,c)) but transparent monolayers (Figure S4) with compromised permeability (Figure 3(a)), a response similar to that observed upon exposure of bacteria-free epithelial-immune cultures to FedS (Figure 2(a,b,e,f)). Interestingly, irregular cell aggregate structures were observed in BEDCC exposed to FedS under hypoxia (Figure 3(c)). These structures showed enhanced F-actin intensity and Ki67 signals indicating proliferative cells. In addition, live cell signals were observed in these structures (Figure 3(d)), indicated some degree of preservation of monolayer viability in the presence of bacteria.

### Intracellular stress was modulated by bile components and bacterial consortium

Intracellular stress, including endoplasmic reticulum (ER) stress and oxidative stress, may contribute to enhanced sensitivity to bile at low oxygen tensions. Both bile exposure and hypoxia are known to induce intracellular stress,^49–52^ which can lead to detrimental cellular effects including DNA damage,^53–56^ ultimately resulting in cell death. In addition, it is reported that commensal bacteria have the ability to alleviate intracellular stress.^57–60^ Thus, we hypothesized that commensal microbes may reduce intracellular stress under hypoxia and during bile exposure. Expression levels of three genes associated with cellular responses to intracellular stress, PDK1, NrF2, and YAP1 were measured using RT-qPCR. Due to extremely low viabilities in hypoxic cultures incorporating FasS but no bacteria, we were unable to collect sufficient mRNA samples to directly assess the impact of bile on gene expression in the absence of bacteria or the impact of incorporating bacteria in the presence of bile. However, comparing gene expression in bacterial co-cultures with and without bile, the elevation of NrF2 expression with FasS exposure suggests a positive adaptation of cells to potential oxidative stress induced by bile components (Figure 3(f)). Further, the incorporation of the consortium significantly down-regulated expression of YAP1, reflecting lowered cellular stress responses from cells.

### Model bile at fasted state concentrations mitigates damage and inflammation induced by co-culture with a bacterial consortium

As noted above, BEDCC FasS maintained barrier function (Figure 3(a–c)). In the absence of bile, however, incorporating a bacterial consortium into the intestinal (epithelial/immune) model and co-culturing under apical hypoxic conditions resulted in loss of barrier function (Figure 3(a)) and extensive damage to the epithelial monolayer visible in most cultures by the 24-h endpoint (Figure 3(b,c)). In addition to mitigating damage, inclusion of bile also modulated the immune response in bacterial co-cultures. The incorporation of the bacterial consortium led to significantly elevated TNF-α and interleukin 10 (IL-10) secretion in the intestinal model (Figure 3(e)). However, inclusion of FasS bile resulted in markedly decreased levels of TNF-α and IL-10 secretion, indicating bile exposure suppressed the bacteria-associated inflammatory response. These results indicate that bile modifies intestinal model tolerance to bacteria, potentially by impacting the response of the host cells as well as bacteria population directly.

### Model bile regulated consortium growth and composition

**Figure 4.**
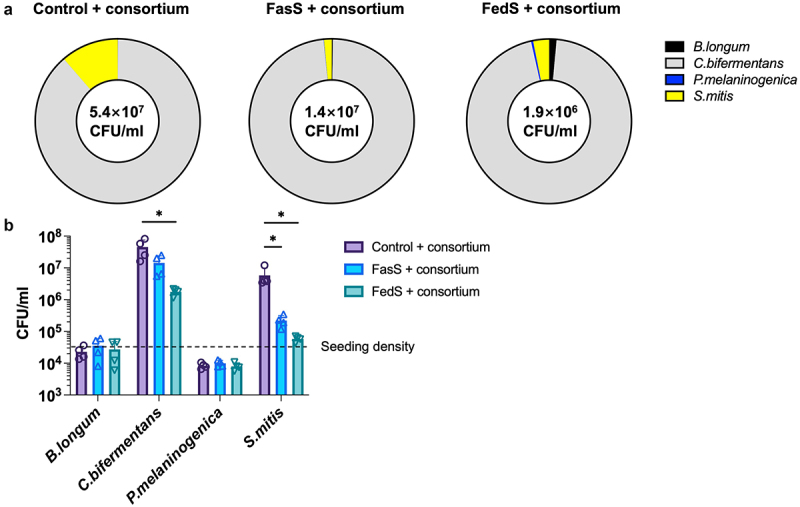
BS/PC model bile micelles modulate consortium communities in BEDCC including (a) total number of microbes in apical compartment, and (b) relative amounts of the bacterial species in the consortium. (Control: No BS/PC). Significant differences from one-way ANOVA analysis: *p < 0.05.

Bile has been reported to have antibiotic properties due to its detergent-like function that disrupts bacterial cell membranes during replication. In this study, strain density measurements using qPCR in media collected from BEDCC revealed lower bacterial numbers in systems incorporating model bile (FasS and FedS), suggesting that bile components created a more bacteriostatic environment *in vitro* compared to culture in the absence of bile (Figure 4(a,b)). A significant effect of bile on levels of *S. mitis*, a commensal bacteria with pathogenic potential,^61,62^ was observed, suggesting bile can selectively supress growth of certain species without significantly impacting others. FedS exposure also negatively impacted the density of *C. bifermentans*. Bacteriostatic effects of model bile may have contributed to the maintenance of epithelial barrier integrity in co-cultures incorporating FasS.

### Mucus layer protects epithelium from model bile in vitro

**Figure 5.**
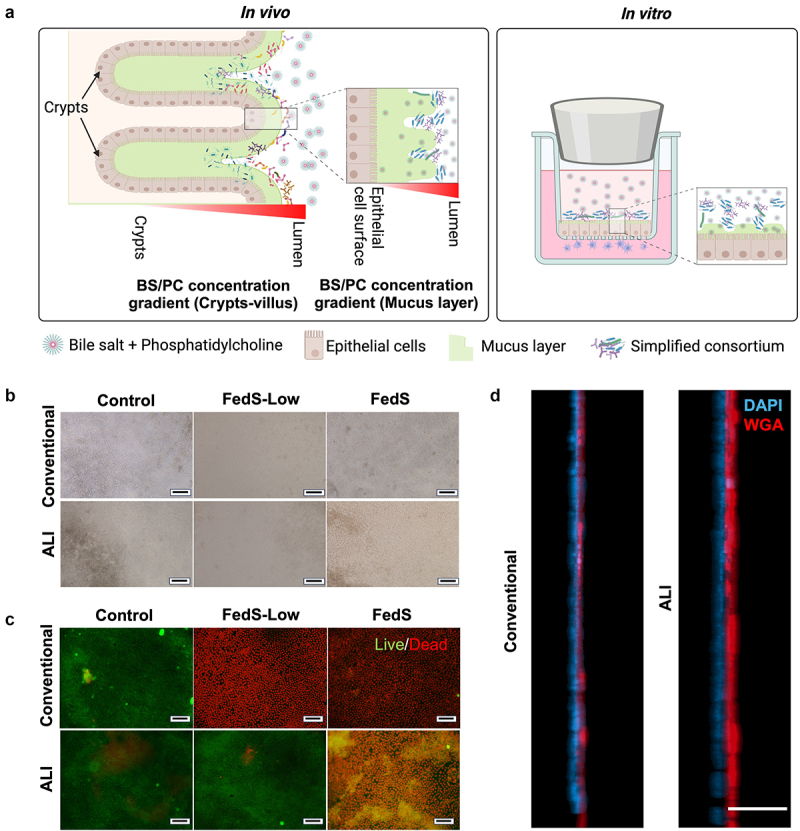
Mucus regulates intestinal model tolerance to bile components. (a) Schematic of the thick mucus layer and crypt-villus architecture in vivo that may impact exposure to high concentrations of bile and modulate bile toxicity to intestinal epithelial cells (left), and relatively thin mucus layer and flat monolayer structure on in vitro Transwell® inserts (right). (b-c) Impact of ALI vs. conventional culture on response to bile components as reflected in b) structure (brightfield microscopy) and (c) viability indicated by live(green)/Dead(red) staining. (d) Mucus layer thickness: ALI vs. conventional culture methods. (**Control**: No BS/PC). Scale bar: b. 200 μm, c. 100 μm, d. 50 μm.

Our results support the significance of phyisologically relevant parameters, including oxygen level and presence of bacteria, in dictating intestinal model susceptibility to damage from bile components. Primary human epithelial cells exhibited limited tolerance to model bile at levels representing the Fed State, however, even with bacterial co-culture. The thicker mucus layer and crypt-villus structure of native intestine (Figure 5(a)), in contrast to the thinner mucus layer and non-physiological flat architecture of the intestinal model presented, could act to extend the distance between luminal bile and cells and generate a bile concentration gradient. Gastrointestinal mucin could interact with BS and PC.^63,64^ Thus, the bile concentrations contacting epithelial cells *in vivo* may be lower than those measured in luminal extracts.^12,41,65^ To test the significance of mucus in protection from bile components, two different culture methods, conventional (submerged apical epithelial surface) and ALI culture, were used, as ALI culture of primary intestinal epithelium has been shown to result in a thicker mucus layer.^39^ These cultures were exposed to FedS or a concentration of bile components between fasted and fed states (FedS-Low) in an aerobic environment. The thickness of the mucus layer was significantly increased with the ALI culture (Figure 5(d)). The thicker mucus layer did result in increased viability of epithelial cells after 24-h exposure to both FedS-Low and FedS model bile, with the increase in viability in the FedS-Low group most notable with most cells alive, and some cells alive with partial monolayer lifting in the cultures exposed to FedS model bile (Figure 5(b,c)).

### Mucus and high fat stimulus affect commensal bacterial behaviors

As mucus was shown to have a protective effect on the epithelial-immune culture, a mucus-enriched ALI cultivated BEDCC was employed to test response to a “high fat” luminal cue. High-fat diet has been shown to alter consortium composition and elevate DGAT2 expression in mice.^37^ OA, a fatty acid commonly utilized to mimic fat-based nutritional interventions, was introduced as a luminal cue. The goal was to assess whether this system could withstand exposure to OA and determine if OA, as a component of fat intake, could regulate the consortium composition and DGAT expression in our system.

**Figure 6.**
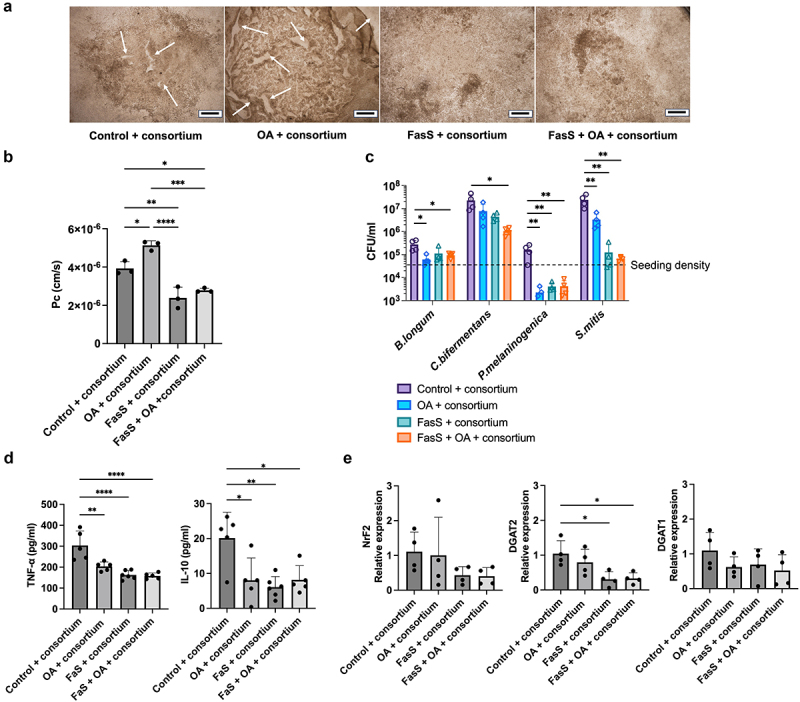
ALI culture and OA intervention effect on monolayer integrity, consortium composition, oxidative stress, and metabolic gene expression. (a) Brightfield images and (b) permeability measurements after 24 h of culture indicate inclusion of BS mitigates impact of consortium co-culture and OA exposure on barrier integrity (lifted area marked with white arrows). (c) Bacterial consortium is maintained over 24 h of culture on ALI monolayer, and relative amounts of bacterial species are modulated by incorporation of model bile and/or OA. (d) immune response to incorporation of bacterial consortium is mitigated by model bile and OA. (e) Oxidative stress is not impacted by incorporation of luminal cues, but epithelial metabolic gene expression is affected by FasS. (**Control**: No BS/PC, No OA) Scale bar: 500 μm. Significant differences from one-way ANOVA analysis: *p < 0.05, **p < 0.01, ***p < 0.001, ****p < 0.0001.

Incorporation of the bacterial consortium into the ALI intestinal-epithelial model in the absence of model bile or OA visibly damaged monolayer integrity (Figure 6(a)), as had been previously observed in the non-ALI intestinal model (Figure 3(b)). Inclusion of OA induced even more severe damage, evidenced by monolayer lifting (Figure 6(a)). However, the inclusion of FasS, either alone or in combination with 50 μM OA, resulted in the preservation of monolayer integrity and alleviation of damage in ALI BEDCC, as evidenced both from microscopic appearance and LY permeability (Figure 6(a,b)), supporting the protective effects of BS/PC during bacterial co-culture observed in non-ALI BEDCC.

Analysis of the bacterial consortium composition following 24 h of ALI BEDCC (Figure 6(c)) in comparison to co-cultures with less mucus (non-ALI BEDCC, Figure 4(b), Figure S5) indicated mucus impacted the relative growth of commensal microbes. In the mucus-enriched ALI BEDCC, the density of *B*. *longum* and *P. melaninogenica* increasing by nine and five times, respectively, relative to the seeding density over the course of 24 h of hypoxic co-culture. In contrast, the abundance of these strains diminished over culture time in the non-ALI BEDCC with less mucus, suggesting that mucus serves as a crucial environmental factor contributing to the adaptation of these strains. *S. mitis* also grew to a greater extent in the ALI group with more mucus content compared to the non-ALI BEDCC (Figure S5). Overall, the trend of bacterial abundance alteration with bile exposure in ALI BEDCC was similar to that of the non-ALI cultures. *S. mitis* experienced a significant decrease in abundance with the inclusion of bile. *C. bifermentans* had a wide density variation but decreased five times on average. The density of *P. melaninogenica* also decreased significantly after BS/PC exposure, a trend only observed in the mucus-enriched ALI BEDCC. The incorporation of OA alone into ALI BEDCC reduced the abundance of all strains, although the effect was not statistically significant for *C. bifermentans* due to high variability. This may be due to the antibiotic function of OA.^66^ OA has been reported to destabilize and lyse bacterial membranes and inhibit membrane-associated enzymes on bacteria.^67^ In contrast, *Clostridium* strain abundance was reported to increase in mice fed high fat diet.^37^ Notably, incorporating OA into ALI BEDCC in the presence of model bile did not have a significant impact on abundance of any bacterial strains.

Interestingly, secretion of TNF-α and IL-10 from ALI BEDCC was reduced relative to conventional (non-ALI) BEDCC (Figure 6(d), Figure S6). This lower secretion may be related to the greater levels of secreted mucus in the ALI BEDCC, as mucus can reduce bacteria-host contact,^39^ and modulate the host immune responses.^68,69^ Further, the secretion of TNF-α and IL-10 by ALI BEDCC was reduced with inclusion of BS/PC and OA (Figure 6(d)), similar to reduced secretion with inclusion of BS/PC in conventional (non-ALI) BEDCC (Figure 3(e)). As inclusion of BS/PC and OA also reduced bacteria number, the lower levels of TNF-α and IL-10 may be due to less microbe exposure and thus less immune response to the bacteria.

The expression levels of Nrf2, DGAT1, and DGAT2 were assessed to gauge oxidative stress adaptation and metabolic activities (Figure 6(e)). In contrast to measured expression in conventional (non-ALI) BEDCC (Figure 3(d)), there were no significant differences in Nrf2 expression among the mucus-enriched ALI BEDCC groups following exposure to model bile and OA, indicating that mucus may help alleviate effects caused by bile components and OA. Additionally, exposure to FasS or FasS + OA significantly lowers the expression of DGAT2. The parallel trend of decreasing density of *C. bifermentans* aligns with previous reports which indicated the metabolites (conditioned medium) of *C. bifermentans* regulate DGAT2 expression.^37^ The expression of DGAT1 was not affected by exposure to FasS or OA.

## Discussion

In recent decades, increasing evidence suggests that bile plays a crucial role in maintaining intestinal homeostasis. Bile has been shown to stimulate epithelial stem cell proliferation and epithelial cell antimicrobial peptide secretion,^70^ suppress bacterial growth, regulate cell apoptosis,^21,71,72^ and alleviate inflammatory responses.^16,73^ BA have also emerged as promising candidates for intestinal disease therapy, owing not only to their well-documented antimicrobial properties,^5^ but also to their affinity for various receptors on cell membranes or within cell nuclei, allowing them to regulate essential cellular activities.^9–11,74^

Most studies to date of bile salt impact on intestinal function were conducted in animal models or using cancer cell lines^21,31,75^ which lack key features of human biology. Moreover, while BA like TCA, GCDCA, and TUDCA have shown beneficial effects in the gastrointestinal (GI) tract, elevated concentrations of BA are reported to be toxic to intestinal cells *in vivo*^1^ and *in vitro*.^76^ Toxicity to intestinal cells *in vitro* is a major hurdle in the incorporation of bile into intestinal models. Threshold concentrations at which BA induce toxicity in *in vitro* primary human cell studies are not well defined, in particular, with regard to the formulation of multiple BS intended to mimic native bile content. While valuable insight has been gained from *in vitro* studies to date, the majority of reported *in vitro* bile experiments have primarily focused on exploring simple interactions, such as BS–epithelial cell interactions^19,21,77–79^ and BA–bacteria interactions.^4,80^ Models consisting of cultures on Transwell® permeable supports have been widely used in BA-intestinal interaction experiments. These studies have demonstrated various regulatory roles of bile in orchestration of processes including intestinal epithelial monolayer repair,^78^ inflammatory response,^81^ and disruption of epithelium integrity in response to high concentrations of deconjugated BA.^21^

The human upper GI system is complex, with bacteria-epithelium-immune cross-talk and its modulation by bile translating to important effects on human health. Development of intestinal models capturing key features of this cross-talk may result in robust platforms offering novel insight for drug screening and nutritional intervention studies. In this study, we present successful development of an *in vitro* human intestinal model that incorporates a physiologically relevant bile formulation consisting of NaTC, NaGCDC, NaTUDC, and PC. This model incorporates primary human intestinal epithelial cells, dendritic cells, and a consortium of commensal bacterial strains to investigate the effects of bile in a physiologically relevant context. We systematically manipulated key factors including model bile component concentrations, bacterial inoculation, oxygen concentration, and mucus thickness. The goal was to discern how these factors contribute to overall model homeostasis as reflected in monolayer integrity (permeability and viability), inflammatory responses, and bacterial growth.

Oxygen level is a critical but often ignored factor in *in vitro* gastrointestinal studies. The lumen of the upper gastrointestinal (GI) tract is characterized as a hypoxic environment.^82^ Cell activities are affected by low oxygen level. For instance, when exposed to a hypoxic apical environment, human intestinal epithelial cells were reported to alter expression of hypoxia-inducible factor target genes, indicating cells adapting to the low oxygen environment.^34^ Under hypoxia, cells have a higher stress status, including the development of ER stress and increased accumulation of reactive oxygen species (ROS), which leads to oxidative stress.^83,84^ ER stress and ROS accumulation can result in DNA damage, and other detrimental effects.^55,85^ In our system, a design intended to create hypoxia was employed. This design uses a stopper fitting into the apical compartment to limit oxygen availability and expose cells to a low oxygen environment. BA have been reported to elevate intracellular ROS levels *in vitro*.^76,86^ Thus, primary human intestinal epithelial cells in hypoxic conditon may have had greater ER and oxidative stress relative to aerobic culture, which was potentially exacerbated with exposure to BS, resulting in low cell viability (Figure 2(d,h)).

Expression levels of genes associated with hypoxia or intracellular stress, PDK1, Nrf2, and YAP1, were analyzed to assess the proposed significance of oxidative stress in response to BS under hypoxia. PDK1, a kinase enzyme, plays a role in inactivating the enzyme pyruvate dehydrogenase and is directly trans-activated by HIF-α, so it is a marker of HIF-α activation.^87,88^ Nrf2, a transcription factor, regulates antioxidant genes responsible for producing superoxide dismutase or glutathione peroxidase.^89,90^ Its pathway is known to be affected by ROS-mediated intracellular oxidative stress.^91^ YAP1 regulates the Hippo pathway, which in turn regulates cell proliferation and apoptosis.^92^ It can be activated via ER stress and ROS damage, and thus elevation of its expression may serve as a sign of damage caused by these intracellular stresses.^93,94^ Our study demonstrated the up-regulation of Nrf2 gene expression in BEDCC exposed to FasS compared to BEDCC not exposed to FasS, indicating cells may adapt to the possible high oxidative stress under exposure to hypoxia and model bile (Figure 3(f)). However, it should be noted that the data only suggest that cell stress may be a contributing mechanism affecting cell viability during bile exposure and bacterial co-culture. Direct oxidative stress measurement, like ROS quantification and analysis of antioxidant gene expressions,^95^ should be conducted in future studies. Down-regulation of YAP1 expression when microbes were inoculated in the absence of model bile suggests alleviation of cellular stress by the consortium. Unfortunately, direct assessment of the impact of the bacterial consortium on gene expression in the presence of model bile was not possible since there was significant damage with exposure to bile under hypoxia in the absence of bacteria, precluding collection of adequate genetic material. The observation that a commensal bacterial consortium can mitigate the toxicity of model bile under hypoxia (Figures 2h, 3d) is one of the most significant in this study, and underlying mechanisms should be explored.

Another significant finding of this study is the contribution of model bile to maintenance of homeostasis upon introduction of the bacterial consortium in the BEDCC model, as reflected in barrier integrity, viability, and decreased inflammatory response (Figure 3(a–e)). In addition to its impact on host cell viability and function, bile has been recognized for its diverse antimicrobial properties, varying based on the type and strains of bacteria. This study demonstrated that bile at physiologically relevant concentrations regulated consortium composition as reflected in relative levels of strains and created a more bacteriostatic environment (Figure 4). *S. mitis*, which is reported to be in the human duodenum and human oral cavity,^36,96^ exhibited heightened sensitivity to bile in our experiments. Upon bile exposure, *S. mitis* apparently ceased growth, and density of organisms dropped significantly below the seeding density over 24 h of culture. *C. bifermentans* ATCC 638, a microbe that is significant in intestinal lipid metabolism,^37^ dominated the bacterial population and increased in numbers from the time of seeding even with bile exposure, bile did apparently slow proliferation. In contrast, *B. longum*, a probiotic species in the intestine that is resistant to BA^97^ and has BSH activity reported for many member strains.^98^ In this study, it did not grow but maintained viability at around the seeding concentration level in both the presence and absence of bile, possibly due to lack of significant mucus production,^99^ or the dominance of *C. bifermentans* or *S. mitis* that consumed most of the available nutrients. Finally, *P. melaninogenica*, another commensal strain found in the human duodenum,^36^ demonstrated limited growth in absence of bile with no statistically significant response to bile exposure. Overall, all strains remained viable, with growth either not impacted or inhibited by bile exposure.

Recognizing the influence of commensal microbes on bile tolerance, we sought to explore other physiological factors that may contribute to tolerance and homeostasis in the duodenal lumen *in vivo*^41^ yet are missing *in vitro*. Noting that mucus forms a crucial barrier between intestinal lumen contents and underlying tissues,^100^ and that mucus production *in vitro* is generally markedly less than occurs *in vivo*, we sought to evaluate the ability of mucus to buffer response to bile in the model intestinal system. A reported method^39^ including ALI culture and supplementation with VIP was used to generate a thicker mucus layer relative to conventional culture methods, increasing average thickness from approximately 4 μm to 13 μm (Figure 5(d)). Monolayers with a thicker mucus layer demonstrated resistance to FedS-Low exposure, maintaining greater than 95% viability in aerobic culture. In contrast, conventional cultures could not tolerate FedS-Low exposure (Figure 5(c)). Further, Nrf2 expression did not increase significantly with exposure to bile (Figure 6(e)), as had been observed in conventional (non-ALI) BEDCC (Figure 3(f)), possibly due to bile effects being alleviated by mucus. These findings support a significant role of the mucus layer in buffering bile toxicity to the intestinal epithelium. The actual extracellular concentration of bile at the apical surface of the small intestinal epithelium may be lower than the values measured in fluid collected from the intestinal lumen.

Interestingly, we observed distinct behavior of the consortia on mucus-enriched ALI relative to conventional epithelial-immune cultures. *B. longum* and *P. melaninogenica*, known for possessing mucus-metabolizing genes,^101,102^ exhibited expansion in ALI BEDCC in the absence of BS/PC, unlike in conventional culture groups where they remained viable but did not grow over the 24-h culture period (Figures 4b, 6c and S5). Similarly, *S. mitis* exhibited a higher growth in the ALI group compared to conventional culture groups, which can be explained by the ability of *S. mitis* to utilize mucin as a carbon source.^103^ Conversely, *C. bifermentans*, lacking reported mucus-degrading capacity,^104,105^ did not exhibit enhanced growth in the ALI groups. Overall, these results suggest that mucus serves not only as a structural barrier to reduce bile toxicity to the epithelium but also as an important environmental factor facilitating the adaptation of commensal microbes with mucus-degrading functions. Inclusion of model bile in ALI BEDCC enabled maintenance of monolayer confluency with the incorporation of the bacterial consortium as in conventional culture. Interestingly, the bacteriostatic effect of model bile was similar, or greater for some strains in ALI relative to conventional cultures, suggesting the presence of mucus did not hinder bacteriostatic effects of model bile and in fact possibly acted synergistically. It is worth noting that while bacteriostatic effects of OA in the ALI BEDCC model were overall similar in magnitude to those of model bile, only inclusion of model bile enabled maintenance of monolayer confluency and barrier function with bacterial co-culture, suggesting bile components may have directly impacted the epithelial-immune model’s response to bacteria.

In this study, the inoculation of bacteria stimulated both pro- and anti-inflammatory cytokine secretion in the immune-competent intestinal model. The introduction of model bile in the presence of bacteria reduced the secretion of TNF-α and IL-10. This observation aligns with reported data indicating that bile can mitigate inflammatory responses by decreasing TNF-α secretion.^13^ However, the reduction of IL-10 secretion contradicts reported data.^16^ The simultaneous suppression of both pro-inflammatory and anti-inflammatory cytokines could be attributed to the alleviation of original inflammatory stimulation caused by the consortium. Total bacterial units declined from 5.4 × 10^7^ CFU/mL (No BS/PC) to 1.4 × 10^7^ CFU/mL (FasS) and 1.9 × 10^6^ CFU/mL (FedS) in the presence of bile, indicating the causative factor of the inflammatory response was suppressed (Figure 4(a)). These data indicate that model bile may contribute to gut homeostasis by regulating bacterial abundance, thereby alleviating cell inflammatory responses. However, it is also possible that bile directly regulates dendritic cells, as reported in previous studies.^33,106^ Since adding BS/PC to the epithelial cells without inducing cytotoxicity in the absence of bacteria was not possible, future studies utilizing isolated immune cell with BS/PC and pro-inflammatory stimuli, such as bacterial spent medium or heat-killed bacteria, could provide further supporting insight.

When ALI culture was used to increase mucus thickness, the secretion levels of both TNF- α and IL-10 were lower in bacterial co-culture in the absence of model bile (and IL-10 was also lower in the presence of model bile) compared to conventional culture (Figure S6). This result aligns with another report that mucus can delay or eliminate the immune response triggered by bacterial infection,^39^ suggesting that host-derived mucus plays a multifaceted role: not only supporting the growth of certain strains but also mitigating bacterial-induced immune responses.

In an initial attempt to explore this mucosal model’s ability to capture response to a nutritional cue, the BEDCC-ALI system was dosed apically with OA to mimic a “high fat cue”. Previous studies on rodents have noted that bacterial shifts including increases in *Clostridiaceae* and decreases in *Bifidobacteriaceae* and *Bacteroidaceae* as well as increased lipid absorption are hallmarks of response to high fat diet.^37^ However, exposure to OA (mimicking fat intervention) in the absence of model bile reduced the density of all four strains, possibly due to its reported antibiotic functions,^66^ while exposure to OA in the presence of model bile did not significantly impact density of any strains, although a decreasing trend of *C. bifermentans* was observed. Under FasS or OA + FasS treatment, expression of DGAT2 (but not DGAT1), was down-regulated. The parallel trend of reduction in *C. bifermentans*, while only correlative and requiring further analysis to establish causality and mechanism, corroborates previous reports^37^ indicating *C. bifermentans* regulates host fat metabolism by elevating the expression of DGAT2 in mice. These results suggest potential changes in lipid trafficking in response to bile components. DGAT2 and DGAT1 are predominantly expressed in mouse and human intestine, respectively, but expression of DGAT2 can play a compensatory role in intestinal cells of DGAT1-deficient human patients.^107^ Studies in mice have indicated complementary roles of DGAT1 and DGAT2 in triacylglycerol distribution into distinct classes of cytosolic lipid droplets with specialized roles in lipid metabolism and transport. Future studies using the BEDCC-ALI system could explore whether altered DGAT expression corresponds with similar changes in lipid trafficking within human cells. Future efforts to employ the BEDCC-ALI in exposure to high fat and other nutritional cues could incorporate more complex and physiologically relevant consortia and/or different types of fatty acids, such as stearic acid and palmitic acid, commonly used in animal studies to simulate high-fat diets.^37^ Incorporation of BEDCC-ALI into a chip-based fluidic platform might facilitate transient exposure to a high fat cue in the context of flow mimicking gastrointestinal transit.

Despite the interesting findings demonstrated by this model, several limitations remain, and future optimizations could enhance its applicability. A primary limitation is the lack of similarity of several model features to physiological conditions. For example, while the BS/PC model employed in this study incorporates multiple BSs and PC to form bile micelles at physiologically relevant concentrations, it lacks other important components such as other bile acids known to be present in human bile,^108,109^ cholesterol,^110^ and fatty acids,^111^ all of which play important roles in bile micelle formation and host–microbiome interactions. Notably, some secondary bile acids are present in human bile, but multiple secondary bile acids were not included in this study as ultimately, we would like to test the production of secondary bile acids as a function of the microbiota in our system. Secondary bile acids including deoxycholic acid^112^ and lithocholic acid^113^ have been reported to markedly influence host physiology. Additionally, cholesterol, a major lipid component of human bile, contributes to micelle formation and host cell functions,^110,114^ while fatty acids, which are abundant in the intestinal lumen – especially in the fed state – modulate host and bacterial activity.^115,116^ However, this study included only oleic acid, limiting the model’s representation of physiological lipid exposure. Future studies should integrate a broader spectrum of bile acids, cholesterol, and physiologically relevant fatty acids to improve the biological relevance and accuracy of the model. Another model feature varying considerably from physiological conditions is the simplified bacterial consortium. Although the four selected strains were chosen based on their presence in the duodenum and key metabolic functions, they do not fully reflect the diversity of the gut microbiota. *S.mitis* and *P.melaninogenica* have been reported with 13.66 ± 11.64% and 5.29 ± 4.02% relative abundance in human duodenum.^36^ However, *Lactobacillus*, a genus abundantly found in the duodenum,^117^ was not included. Further, the four selected strains were not seeded at relative densities that reflect their natural abundance in the human duodenum, in large part due to the lack of available quantitative data. For instance, Clostridium Cluster I, the broader taxonomic group of *C. bifermentans*, has been reported at 0.30 ± 0.78% relative abundance,^36^ while *Bifidobacterium* has been reported at a relative abundance of 0.52 ± 0.60%.^36^ Future refinements should incorporate a more representative microbial consortium, including key taxa from the human gut microbiome at relevant relative abundance, to better capture commensal bacterial influences on intestinal health. While the initial bacterial density of 10^5^ CFU/mL was close to *in vivo* conditions,^118^ bacterial density increased to 10^7^ CFU/mL by the experiment’s endpoint due to the static nature of the Transwell system. Implementing microfluidic-based models in future studies could introduce dynamic flow conditions, would be expected to help regulating bacterial growth and enhance the model’s physiological relevance.^119^ Additionally, the 24-h exposure to a constant bile concentration does not precisely mimic postprandial physiological conditions. Microfluidic systems could facilitate shorter or periodic exposures to mimic fasting and fed states.

While many commensal bacteria, including many *B. longum* strains, are known to utilize bile salts and produce bile metabolites that regulate host cell activity,^98,120^ we did not confirm BSH activity of the specific strain we used in this study. Thus, it is important to determine whether the selected bacterial strains exhibit BSH activity, and, if present, characterize the post-hydrolysis bile composition. If absent, incorporating BSH-positive strains could establish a more physiologically relevant bile pool, improving the model’s fidelity to *in vivo* bacteria-bile-host cross-talk. Finally, as the intestinal epithelial cells used in this study were derived from a single donor (H406), donor specificity must be considered. The possibility of donor-specific cell responses raises concerns regarding the model’s generalizability. Future studies should include cells from multiple donors to account for potential inter-individual variability. Addressing these limitations will contribute to the development of a more physiologically representative in vitro system for studying host-bile-microbiome interactions in the human gut.

In conclusion, despite some limitations, our results support the utility of a human immune-competent small intestinal model capturing key features of the native mucosal environment for studying bile-bacteria-host cross-talk. Using this model, we revealed the sensitivity of primary human intestinal epithelial monolayers generated from duodenal organoids to varying concentrations of model bile. Oxygen was found to be a critical factor affecting bile toxicity to cells. Incorporation of an upper GI-relevant bacterial consortium aided in sustaining intestinal monolayer integrity upon exposure to physiological concentrations of bile mimicking the human fasted state under hypoxia. Further, inclusion of bile mitigated barrier damage and immune response upon incorporation of the bacterial consortium. Bile regulated bacterial community composition and created a more bacteriostatic environment in epithelial-immune co-cultures, potentially aiding in stabilizing the co-culture system via this mechanism. The results suggest that the presence of essential intestinal luminal molecules, such as bile, could contribute to homeostasis in *in vitro* gut-bacterial models. The inclusion of a mucus layer was also shown to support the adaptation of mucus-degrading commensal microbes and contribute to buffering bile toxicity to intestinal cells, indicating the significance of physiological mucus structures in bile-microbial-intestinal *in vitro* studies. Composition of the bacterial consortium was modulated by a high fat stimulus in the absence but not the presence of model bile and model bile modulated DGAT2 expression, suggesting a potential impact on lipid trafficking. Taken together, these results support the use of an immune-competent primary intestinal model for providing novel insight into bile-bacteria-host cross-talk.

## Supplementary Material

Bile salt research _supplemental materials.docx

## Data Availability

The authors confirm that the data supporting the findings of this study are available within the article. Source data associated with this manuscript are available by contacting the corresponding author.

## References

[1] Hofmann AF. The continuing importance of bile acids in liver and intestinal disease. Arch Intern Med. 1999;159(22):2647–25. doi: 10.1001/archinte.159.22.2647. 10597755

[2] Islam KBMS, Fukiya S, Hagio M, Fujii N, Ishizuka S, Ooka T, Ogura Y, Hayashi T, Yokota A. Bile acid is a host factor that regulates the composition of the cecal microbiota in rats. Gastroenterology. 2011;141(5):1773–1781. doi:10.1053/j.gastro.2011.07.046. 21839040

[3] An C, Chon H, Ku W, Eom S, Seok M, Kim S, Lee J, Kim D, Lee S, Koo H, et al. Bile acids: major regulator of the gut microbiome. Microorganisms. 2022;10(9):1792. doi: 10.3390/microorganisms10091792. 36144395 PMC9502002

[4] Sannasiddappa TH, Lund PA, Clarke SR. In vitro antibacterial activity of unconjugated and conjugated bile salts on staphylococcus aureus. Front Microbiol. 2017;8:1581. doi: 10.3389/fmicb.2017.01581. 28878747 PMC5572772

[5] Urdaneta V, Casadesus J. Interactions between bacteria and bile salts in the gastrointestinal and hepatobiliary tracts. Front Med (Lausanne). 2017;4:163. doi: 10.3389/fmed.2017.00163. 29043249 PMC5632352

[6] de Aguiar Vallim TQ, Tarling EJ, Edwards PA. Pleiotropic roles of bile acids in metabolism. Cell Metab. 2013;17(5):657–669. doi: 10.1016/j.cmet.2013.03.013. 23602448 PMC3654004

[7] Dawson PA, Karpen SJ. Intestinal transport and metabolism of bile acids. J Lipid Res. 2015;56(6):1085–1099. doi: 10.1194/jlr.R054114. 25210150 PMC4442867

[8] Calzadilla N, Comiskey SM, Dudeja PK, Saksena S, Gill RK, Alrefai WA. Bile acids as inflammatory mediators and modulators of intestinal permeability. Front Immunol. 2022;13. doi: 10.3389/fimmu.2022.1021924. PMC9768584 36569849

[9] Ticho AL, Malhotra P, Dudeja PK, Gill RK, Alrefai WA. Bile acid receptors and gastrointestinal functions. Liver Res. 2019;3(1):31–39. doi: 10.1016/j.livres.2019.01.001. 32368358 PMC7197881

[10] Sorrentino G, Perino A, Yildiz E, El Alam G, Bou Sleiman M, Gioiello A, Pellicciari R, Schoonjans K. Bile acids signal via TGR5 to activate intestinal stem cells and epithelial regeneration. Gastroenterology. 2020;159(3):956–968 e958. doi: 10.1053/j.gastro.2020.05.067. 32485177

[11] Wahlstrom A, Sayin SI, Marschall HU, Backhed F. Intestinal crosstalk between bile acids and microbiota and its impact on host metabolism. Cell Metab. 2016;24(1):41–50. doi: 10.1016/j.cmet.2016.05.005. 27320064

[12] Perez de la Cruz Moreno M, Oth M, Deferme S, Lammert F, Tack J, Dressman J, Augustijns P. Characterization of fasted-state human intestinal fluids collected from duodenum and jejunum. J Pharm Pharmacol. 2006;58(8):1079–1089. doi: 10.1211/jpp.58.8.0009. 16872555

[13] Yang Y, He J, Suo Y, Lv L, Wang J, Huo C, Zheng Z, Wang Z, Li J, Sun W, et al. Anti-inflammatory effect of taurocholate on TNBS-induced ulcerative colitis in mice. Biomed Pharmacother. 2016;81:424–430. doi: 10.1016/j.biopha.2016.04.037. 27261622

[14] Darkoh C, Brown EL, Kaplan HB, DuPont HL, Ratner AJ. Bile salt inhibition of host cell damage by Clostridium Difficile toxins. PLOS ONE. 2013;8(11):e79631. doi: 10.1371/journal.pone.0079631. 24244530 PMC3823588

[15] Sung JY, Shaffer EA, Costerton JW. Antibacterial activity of bile salts against common biliary pathogens. Digestive Dis Sci. 1993;38(11):2104–2112. doi: 10.1007/BF01297092. 8223087

[16] Sun R, Xu C, Feng B, Gao X, Liu Z. Critical roles of bile acids in regulating intestinal mucosal immune responses. Therap Adv Gastroenterol. 2021;14. doi: 10.1177/17562848211018098. PMC8165529 34104213

[17] Wilson A, Almousa A, Teft WA, Kim RB. Attenuation of bile acid-mediated FXR and PXR activation in patients with Crohn’s disease. Sci Rep. 2020;10(1):1866. doi: 10.1038/s41598-020-58644-w. 32024859 PMC7002620

[18] Inagaki T, Moschetta A, Lee Y-K, Peng L, Zhao G, Downes M, Yu RT, Shelton JM, Richardson JA, Repa JJ, et al. Regulation of antibacterial defense in the small intestine by the nuclear bile acid receptor. Proc Natl Acad Sci USA. 2006;103(10):3920. doi: 10.1073/pnas.0509592103. 16473946 PMC1450165

[19] Laukens D, Devisscher L, Van den Bossche L, Hindryckx P, Vandenbroucke RE, Vandewynckel YP, Cuvelier C, Brinkman BM, Libert C, Vandenabeele P, et al. Tauroursodeoxycholic acid inhibits experimental colitis by preventing early intestinal epithelial cell death. Lab Investigation. 2014;94(12):1419–1430. doi: 10.1038/labinvest.2014.117. 25310532

[20] Perez MJ, Briz O. Bile-acid-induced cell injury and protection. World J Gastroenterol. 2009;15(14):1677–1689. doi: 10.3748/wjg.15.1677. 19360911 PMC2668773

[21] Li DK, Chaudhari SN, Lee Y, Sojoodi M, Adhikari AA, Zukerberg L, Shroff S, Barrett SC, Tanabe K, Chung RT, et al. Inhibition of microbial deconjugation of micellar bile acids protects against intestinal permeability and liver injury. Sci Adv. 2022;8(34):eabo2794. doi: 10.1126/sciadv.abo2794. 36026454 PMC9417178

[22] Barrios JM, Lichtenberger LM. Role of biliary phosphatidylcholine in bile acid protection and NSAID injury of the ileal mucosa in rats. Gastroenterology. 2000;118(6):1179–1186. doi: 10.1016/S0016-5085(00)70371-4. 10833493

[23] Komichi D, Tazuma S, Nishioka T, Hyogo H, Une M, Chayama K. Unique inhibition of bile salt-induced apoptosis by Lecithins and cytoprotective bile salts in immortalized mouse cholangiocytes. Digestive Dis Sci. 2003;48(12):2315–2322. doi: 10.1023/B:DDAS.0000007869.67105.27. 14714619

[24] Li J, Dawson PA. Animal models to study bile acid metabolism. Biochimica et Biophysica Acta (BBA) - Mol Basis Dis. 2019;1865(5):895–911. doi: 10.1016/j.bbadis.2018.05.011. PMC6242766 29782919

[25] Russell DW. The enzymes, regulation, and genetics of bile acid synthesis. Annu Rev Biochem. 2003;72(1):137–174. doi: 10.1146/annurev.biochem.72.121801.161712. 12543708

[26] Govindarajan K, MacSharry J, Casey PG, Shanahan F, Joyce SA, Gahan CG, Makishima M. Unconjugated bile acids influence expression of circadian genes: a potential mechanism for microbe-host crosstalk. PLOS ONE. 2016;11(12):e0167319. doi: 10.1371/journal.pone.0167319. 27907092 PMC5132238

[27] Raimondi F, Santoro P, Barone MV, Pappacoda S, Barretta ML, Nanayakkara M, Apicella C, Capasso L, Paludetto R. Bile acids modulate tight junction structure and barrier function of Caco-2 monolayers via EGFR activation. Am J Physiol Gastrointestinal Liver Physiol. 2008;294(4):G906–913. doi: 10.1152/ajpgi.00043.2007. 18239063

[28] Hagiwara Y, Kumagai H, Ouwerkerk N, Gijzen L, Annida R, Bokkers M, van Vught R, Yoshinari K, Katakawa Y, Motonaga K, et al. A novel in vitro membrane permeability methodology using three-dimensional Caco-2 tubules in a microphysiological system which better mimics in vivo physiological conditions. J Pharm Sci. 2022;111(1):214–224. doi: 10.1016/j.xphs.2021.11.016. 34838780

[29] Pérez-González C, Ceada G, Greco F, Matejčić M, Gómez-González M, Castro N, Menendez A, Kale S, Krndija D, Clark AG, et al. Mechanical compartmentalization of the intestinal organoid enables crypt folding and collective cell migration. Nat Cell Biol. 2021;23(7):745–757. doi: 10.1038/s41556-021-00699-6. 34155382 PMC7611697

[30] Cai J, Sun L, Gonzalez FJ. Gut microbiota-derived bile acids in intestinal immunity, inflammation, and tumorigenesis. Cell Host Microbe. 2022;30(3):289–300. doi: 10.1016/j.chom.2022.02.004. 35271802 PMC8923532

[31] Nunez-Sanchez MA, Herisson FM, Keane JM, Garcia-Gonzalez N, Rossini V, Pinhiero J, Daly J, Bustamante-Garrido M, Hueston CM, Patel S, et al. Microbial bile salt hydrolase activity influences gene expression profiles and gastrointestinal maturation in infant mice. Gut Microbes. 2022;14(1):2149023. doi: 10.1080/19490976.2022.2149023. 36420990 PMC9704388

[32] Jia W, Xie G, Jia W. Bile acid–microbiota crosstalk in gastrointestinal inflammation and carcinogenesis. Nat Rev Gastroenterol Hepatol. 2018;15(2):111–128. doi: 10.1038/nrgastro.2017.119. 29018272 PMC5899973

[33] Hu J, Wang C, Huang X, Yi S, Pan S, Zhang Y, Yuan G, Cao Q, Ye X, Li H. Gut microbiota-mediated secondary bile acids regulate dendritic cells to attenuate autoimmune uveitis through TGR5 signaling. Cell Rep. 2021 [acccessed 2024 05 22]. 36(12):109726. doi: 10.1016/j.celrep.2021.109726. 34551302

[34] Singhal R, Shah YM. Oxygen battle in the gut: hypoxia and hypoxia-inducible factors in metabolic and inflammatory responses in the intestine. J Biol Chem. 2020;295(30):10493–10505. doi: 10.1074/jbc.REV120.011188. 32503843 PMC7383395

[35] Kakni P, Jutten B, Teixeira Oliveira Carvalho D, Penders J, Truckenmuller R, Habibovic P, Giselbrecht S. Hypoxia-tolerant apical-out intestinal organoids to model host-microbiome interactions. J Tissue Eng. 2023;14:20417314221149208. doi: 10.1177/20417314221149208. 36699634 PMC9869231

[36] Cheng J, Kalliomäki M, Heilig HGHJ, Palva A, Lähteenoja H, de Vos WM, Salojärvi J, Satokari R. Duodenal microbiota composition and mucosal homeostasis in pediatric celiac disease. BMC Gastroenterol. 2013;13:113–113. doi: 10.1186/1471-230X-13-113. 23844808 PMC3716955

[37] Martinez-Guryn K, Hubert N, Frazier K, Urlass S, Musch MW, Ojeda P, Pierre JF, Miyoshi J, Sontag TJ, Cham CM, et al. Small intestine microbiota regulate host digestive and absorptive adaptive responses to dietary lipids. Cell Host & Microbe. 2018;23(4):458–469 e455. doi: 10.1016/j.chom.2018.03.011. 29649441 PMC5912695

[38] Mills S, Yang B, Smith GJ, Stanton C, Ross RP. Efficacy of bifidobacterium longum alone or in multi-strain probiotic formulations during early life and beyond. Gut Microbes. 2023;15(1):2186098. doi:10.1080/19490976.2023.2186098. 36896934 PMC10012958

[39] Wang Y, Kim R, Sims CE, Allbritton NL. Building a thick mucus hydrogel layer to improve the physiological relevance of in vitro primary colonic epithelial models. Cellular Mol Gastroenterol Hepatol. 2019;8(4):653–655 e655. doi: 10.1016/j.jcmgh.2019.07.009. PMC6889783 31356887

[40] Luchan J, Choi C, Carrier RL. Reactive oxygen species limit intestinal mucosa-bacteria homeostasis in vitro. Sci Rep. 2021;11(1):23727. doi: 10.1038/s41598-021-02080-x. 34887444 PMC8660821

[41] Riethorst D, Mols R, Duchateau G, Tack J, Brouwers J, Augustijns P. Characterization of human duodenal fluids in fasted and fed state conditions. J Pharm Sci. 2016;105(2):673–681. doi: 10.1002/jps.24603. 26228456

[42] Dahlgren D, Venczel M, Ridoux JP, Skjöld C, Müllertz A, Holm R, Augustijns P, Hellström PM, Lennernäs H. Fasted and fed state human duodenal fluids: characterization, drug solubility, and comparison to simulated fluids and with human bioavailability. Eur J Pharm Biopharm. 2021;163:240–251. doi: 10.1016/j.ejpb.2021.04.005. 33872761

[43] Collado MC, Donat E, Ribes-Koninckx C, Calabuig M, Sanz Y. Imbalances in faecal and duodenal Bifidobacterium species composition in active and non-active coeliac disease. BMC Microbiol. 2008;8(1):232. doi:10.1186/1471-2180-8-232. 19102766 PMC2635381

[44] Kerckhoffs AP, Samsom M, van der Rest ME, de Vogel J, Knol J, Ben-Amor K, Akkermans LM. Lower Bifidobacteria counts in both duodenal mucosa-associated and fecal microbiota in irritable bowel syndrome patients. World J Gastroenterol. 2009;15(23):2887–2892. doi: 10.3748/wjg.15.2887. 19533811 PMC2699007

[45] Sasaki N, Miyamoto K, Maslowski KM, Ohno H, Kanai T, Sato T. Development of a scalable co-culture system for gut anaerobes and human colon epithelium. Gastroenterology. 2020;159(1):388–390.e5. doi: 10.1053/j.gastro.2020.03.021. 32199883

[46] Mikami Y, Grubb BR, Rogers TD, Dang H, Asakura T, Kota P, Gilmore RC, Okuda K, Morton LC, Sun L, et al. Chronic airway epithelial hypoxia exacerbates injury in muco-obstructive lung disease through mucus hyperconcentration. Sci Transl Med. 2023;15(699):eabo7728. doi: 10.1126/scitranslmed.abo7728. 37285404 PMC10664029

[47] Hossain S, Joyce P, Parrow A, Jõemetsa S, Höök F, Larsson P, Bergström CAS. Influence of bile composition on membrane incorporation of transient permeability enhancers. Mol Pharm. 2020;17(11):4226–4240. doi:10.1021/acs.molpharmaceut.0c00668. 32960068 PMC7610231

[48] Grant J, Lee E, Almeida M, Kim S, LoGrande N, Goyal G, Sesay AM, Breault DT, Prantil-Baun R, Ingber DE. Establishment of physiologically relevant oxygen gradients in microfluidic organ chips. Lab Chip. 2022;22(8):1584–1593. doi: 10.1039/D2LC00069E. 35274118 PMC9088163

[49] Cai S-Y, Ouyang X, Chen Y, Soroka CJ, Wang J, Mennone A, Wang Y, Mehal WZ, Jain D, Boyer JL. Bile acids initiate cholestatic liver injury by triggering a hepatocyte-specific inflammatory response. JCI Insight. 2017;2(5). doi: 10.1172/jci.insight.90780. PMC5333973 28289714

[50] Lechner S, Müller-Ladner U, Schlottmann K, Jung B, McClelland M, Rüschoff J, Welsh J, Schölmerich J, Kullmann F. Bile acids mimic oxidative stress induced upregulation of thioredoxin reductase in colon cancer cell lines. Carcinogenesis. 2002 [acccessed 2024 2 28];23(8):1281–1288. doi: 10.1093/carcin/23.8.1281. 12151345

[51] Liu Y, Wang C, Wang Y, Ma Z, Xiao J, McClain C, Li X, Feng W. Cobalt chloride decreases fibroblast growth factor-21 expression dependent on oxidative stress but not hypoxia-inducible factor in caco-2 cells. Toxicol Appl Pharm. 2012;264(2):212–221. doi:10.1016/j.taap.2012.08.003. PMC3593348 22917661

[52] D’Aiuto N, Hochmann J, Millán M, Di Paolo A, Bologna-Molina R, Sotelo Silveira J, Arocena M. Hypoxia, acidification and oxidative stress in cells cultured at large distances from an oxygen source. Sci Rep. 2022;12(1):21699. doi: 10.1038/s41598-022-26205-y. 36522457 PMC9755289

[53] Yamamori T, Meike S, Nagane M, Yasui H, Inanami O. ER stress suppresses DNA double-strand break repair and sensitizes tumor cells to ionizing radiation by stimulating proteasomal degradation of Rad51. FEBS Lett. 2013;587(20):3348–3353. doi:10.1016/j.febslet.2013.08.030. 24021650

[54] Chong WC, Shastri MD, Eri R. Endoplasmic reticulum stress and oxidative stress: a vicious nexus implicated in bowel disease pathophysiology. Int J Mol Sci. 2017;18(4). doi: 10.3390/ijms18040771. PMC5412355 28379196

[55] Pereira C, Gracio D, Teixeira JP, Magro F. Oxidative stress and DNA damage: implications in inflammatory bowel disease. Inflamm Bowel Dis. 2015;21(10):2403–2417. doi: 10.1097/MIB.0000000000000506. 26193347

[56] Kim YJ, Kim EH, Hahm KB. Oxidative stress in inflammation-based gastrointestinal tract diseases: challenges and opportunities. J Gastro Hepatol. 2012;27(6):1004–1010. doi:10.1111/j.1440-1746.2012.07108.x. 22413852

[57] Mo C, Lou X, Xue J, Shi Z, Zhao Y, Wang F, Chen G. The influence of Akkermansia muciniphila on intestinal barrier function. Gut Pathog. 2024;16(1):41. doi:10.1186/s13099-024-00635-7. 39097746 PMC11297771

[58] Wang G, Zhang C, Jiang F, Zhao M, Xie S, Liu X. NOD2-RIP2 signaling alleviates microglial ROS damage and pyroptosis via ULK1-mediated autophagy during Streptococcus pneumonia infection. Neurosci Lett. 2022;783:136743. doi: 10.1016/j.neulet.2022.136743. 35716964

[59] Levy A, Stedman A, Deutsch E, Donnadieu F, Virgin HW, Sansonetti PJ, Nigro G. Innate immune receptor NOD2 mediates LGR5^+^ intestinal stem cell protection against ROS cytotoxicity via mitophagy stimulation. Proc Natl Acad Sci, India, Sect B Biol Sci. 2020;117(4):1994–2003. doi: 10.1073/pnas.1902788117. PMC6994981 31919280

[60] Zhao L, Wang S, Dong J, Shi J, Guan J, Liu D, Liu F, Li B, Huo G. IdentificatiIdentificatiOn, characterization, and antioxidant potential of bifidobacterium longum subsp. longum strains isolated from feces of healthy infants. Front Microbiol. 2021;12:756519. doi: 10.3389/fmicb.2021.756519. 34795651 PMC8593421

[61] Monk M, Patel NR, Elshaboury R, Kubiak DW, Hammond SP. Risk of infective endocarditis in streptococcus mitis bloodstream infections among patients with neutropenia from hematologic malignancies. Open Forum Infect Dis. 2024 [acccessed 2024 11 4];11(3). doi: 10.1093/ofid/ofae063. PMC10917222 38449919

[62] Harth-Chu EN, Alves LA, Theobaldo JD, Salomão MF, Höfling JF, King WF, Smith DJ, Mattos-Graner RO. PcsB expression diversity influences on streptococcus mitis phenotypes associated with host persistence and virulence. Front Microbiol. 2019;10. DOI: 10.3389/fmicb.2019.02567. PMC6861525 31798545

[63] Wiedmann TS, Liang W, Herrington H. Interaction of bile salts with gastrointestinal mucins. Lipids. 2004;39(1):51–58. doi:10.1007/s11745-004-1201-y. 15055235

[64] Wiedmann TS, Liang W, Herrington H. Excluded volume effect of rat intestinal mucin on taurocholate/phosphatidylcholine mixed micelles. J Colloid And Interface Sci. 2004;270(2):321–328. doi:10.1016/j.jcis.2003.09.040. 14697697

[65] Jacobs JP, Dong TS, Agopian V, Lagishetty V, Sundaram V, Noureddin M, Ayoub WS, Durazo F, Benhammou J, Enayati P, et al. Microbiome and bile acid profiles in duodenal aspirates from patients with liver cirrhosis: the microbiome, microbial markers and liver disease study. Hepatol Res. 2018;48(13):1108–1117. doi: 10.1111/hepr.13207. 29923681 PMC6334634

[66] Casillas-Vargas G, Ocasio-Malavé C, Medina S, Morales-Guzmán C, Del Valle RG, Carballeira NM, Sanabria-Ríos DJ. Antibacterial fatty acids: an update of possible mechanisms of action and implications in the development of the next-generation of antibacterial agents. Prog In Lipid Res. 2021;82:101093. doi: 10.1016/j.plipres.2021.101093. 33577909 PMC8137538

[67] Yoon BK, Jackman JA, Valle-González ER, Cho N-J. Antibacterial free fatty acids and monoglycerides: biological activities, experimental testing, and therapeutic applications. Int J Mol Sci. 2018;19(4):1114. doi: 10.3390/ijms19041114. 29642500 PMC5979495

[68] Sheng YH, Hasnain SZ. Mucus and Mucins: the underappreciated host defence system. Front Cell Infect Microbiol. 2022;12. doi: 10.3389/fcimb.2022.856962. PMC9238349 35774401

[69] Johansson MEV, Hansson GC. Immunological aspects of intestinal mucus and mucins. Nat Rev Immunol. 2016;16(10):639–649. doi: 10.1038/nri.2016.88. 27498766 PMC6435297

[70] Tremblay S, Romain G, Roux M, Chen X-L, Brown K, Gibson Deanna L, Ramanathan S, Menendez A. Bile acid administration elicits an intestinal antimicrobial program and reduces the bacterial burden in two mouse models of enteric infection. Infect Immun. 2017 [acccessed 2023 11 19];85(6). doi: 10.1128/iai.00942-16. PMC5442623 28348052

[71] Ignacio Barrasa J, Olmo N, Perez-Ramos P, Santiago-Gomez A, Lecona E, Turnay J, Antonia Lizarbe M. Deoxycholic and chenodeoxycholic bile acids induce apoptosis via oxidative stress in human colon adenocarcinoma cells. Apoptosis. 2011;16(10):1054–1067. doi:10.1007/s10495-011-0633-x. 21789651

[72] Perrone EE, Chen C, Longshore SW, Okezie O, Warner BW, Sun CC, Alaish SM, Strauch ED. Dietary bile acid supplementation improves intestinal integrity and survival in a murine model. J Pediatr Surg. 2010;45(6):1256–1265. doi: 10.1016/j.jpedsurg.2010.02.094. 20620329 PMC2904360

[73] Guo C, Xie S, Chi Z, Zhang J, Liu Y, Zhang L, Zheng M, Zhang X, Xia D, Ke Y, et al. Bile acids control inflammation and metabolic disorder through inhibition of NLRP3 inflammasome. Immunity. 2016;45(4):802–816. doi: 10.1016/j.immuni.2016.09.008. 27692610

[74] Wan YY, Sheng L. Regulation of bile acid receptor activity(☆). Liver Res. 2018;2(4):180–185. doi: 10.1016/j.livres.2018.09.008. 32280557 PMC7147511

[75] Van den Bossche L, Borsboom D, Devriese S, Van Welden S, Holvoet T, Devisscher L, Hindryckx P, De Vos M, Laukens D. Tauroursodeoxycholic acid protects bile acid homeostasis under inflammatory conditions and dampens Crohn’s disease-like ileitis. Lab Investigation. 2017;97(5):519–529. doi:10.1038/labinvest.2017.6. 28165466

[76] Araki Y, Katoh T, Ogawa A, Bamba S, Andoh A, Koyama S, Fujiyama Y, Bamba T. Bile acid modulates transepithelial permeability via the generation of reactive oxygen species in the caco-2 cell line. Free Radical Biol & Med. 2005;39(6):769–780. doi:10.1016/j.freeradbiomed.2005.04.026. 16109307

[77] Enright EF, Govindarajan K, Darrer R, MacSharry J, Joyce SA, Gahan CGM. Gut microbiota-mediated bile acid transformations alter the cellular response to multidrug resistant transporter substrates in vitro: focus on P-glycoprotein. Mol Pharm. 2018;15(12):5711–5727. doi: 10.1021/acs.molpharmaceut.8b00875. 30388019

[78] Mroz MS, Lajczak NK, Goggins BJ, Keely S, Keely SJ. The bile acids, deoxycholic acid and ursodeoxycholic acid, regulate colonic epithelial wound healing. Am J Physiol Gastrointestinal Liver Physiol. 2018;314(3):G378–G387. doi: 10.1152/ajpgi.00435.2016. 29351391

[79] Wang Z, Litterio MC, Muller M, Vauzour D, Oteiza PI. (-)-Epicatechin and NADPH oxidase inhibitors prevent bile acid-induced Caco-2 monolayer permeabilization through ERK1/2 modulation. Redox Biol. 2020;28:101360. doi: 10.1016/j.redox.2019.101360. 31677553 PMC6920094

[80] Tian Y, Gui W, Koo I, Smith PB, Allman EL, Nichols RG, Rimal B, Cai J, Liu Q, Patterson AD. The microbiome modulating activity of bile acids. Gut Microbes. 2020;11(4):979–996. doi:10.1080/19490976.2020.1732268. 32138583 PMC7524280

[81] Fathima A, Jamma T. UDCA ameliorates inflammation driven EMT by inducing TGR5 dependent SOCS1 expression in mouse macrophages. Sci Rep. 2024;14(1):24285. doi: 10.1038/s41598-024-75516-9. 39414916 PMC11484976

[82] Zheng L, Kelly CJ, Colgan SP. Physiologic hypoxia and oxygen homeostasis in the healthy intestine. A review in the theme: cellular responses to hypoxia. Am J physiol-Cell Physiol. 2015 [acccessed 2024 09 24];309(6):C350–C360. doi: 10.1152/ajpcell.00191.2015. 26179603 PMC4572369

[83] LeGrand TS, Aw TY. Chronic hypoxia and glutathione-dependent detoxication in rat small intestine. Am J physiol-Gastr L. 1996 [acccessed 2024 Sep 24];270(4):G725–G729. doi: 10.1152/ajpgi.1996.270.4.G725. 8928804

[84] Zeitouni NE, Chotikatum S, von Köckritz-Blickwede M, Naim HY. The impact of hypoxia on intestinal epithelial cell functions: consequences for invasion by bacterial pathogens. Mol Cell Pediatr. 2016;3(1):14. doi: 10.1186/s40348-016-0041-y. 27002817 PMC4803720

[85] Zeeshan HM, Lee GH, Kim HR, Chae HJ. Endoplasmic reticulum stress and associated ROS. Int J Mol Sci. 2016;17(3):327. doi: 10.3390/ijms17030327. 26950115 PMC4813189

[86] Sarathy J, Detloff SJ, Ao M, Khan N, French S, Sirajuddin H, Nair T, Rao MC. The yin and Yang of bile acid action on tight junctions in a model colonic epithelium. Physiol Rep. 2017;5(10):e13294. doi:10.14814/phy2.13294. 28554966 PMC5449568

[87] Kim JW, Tchernyshyov I, Semenza GL, Dang CV. HIF-1-mediated expression of pyruvate dehydrogenase kinase: a metabolic switch required for cellular adaptation to hypoxia. Cell Metab. 2006;3(3):177–185. doi: 10.1016/j.cmet.2006.02.002. 16517405

[88] Wang X, Shen X, Yan Y, Li H. Pyruvate dehydrogenase kinases (PDKs): an overview toward clinical applications. Biosci Rep. 2021;41(4). doi: 10.1042/BSR20204402. PMC8026821 33739396

[89] Jones RM, Mercante JW, Neish AS. Reactive oxygen production induced by the gut microbiota: pharmacotherapeutic implications. Curr Med Chem. 2012;19(10):1519–1529. doi: 10.2174/092986712799828283. 22360484 PMC4269156

[90] Ma Q. Role of Nrf2 in oxidative stress and toxicity. Annu Rev Pharmacol Toxicol. 2013 [acccessed 2023 Nov 19];53(1):401–426. doi: 10.1146/annurev-pharmtox-011112-140320. 23294312 PMC4680839

[91] Buelna-Chontal M, Zazueta C. Redox activation of Nrf2 & NF-kappaB: a double end sword? Cellular Signalling. 2013;25(12):2548–2557. doi: 10.1016/j.cellsig.2013.08.007. 23993959

[92] Zhang L, Song X, Li X, Wu C, Jiang J. Yes-associated protein 1 as a novel prognostic biomarker for gastrointestinal cancer: a meta-analysis. biomed Res Int. 2018;2018:4039173. doi: 10.1155/2018/4039173. 30539010 PMC6261404

[93] Cho Y, Park MJ, Kim K, Kim SW, Kim W, Oh S, Lee JH. Reactive oxygen species-induced activation of Yes-associated protein-1 through the c-Myc pathway is a therapeutic target in hepatocellular carcinoma. World J Gastroenterol. 2020 Nov 14;26(42):2219–2840 (Electronic). 10.3748/wjg.v26.i42.6599. PMC7673967 33268949

[94] Gao Y, Wei H, Peng X, Wang C, Zhu H, Yin J. ER stress-induced YAP upregulation leads to chondrocyte phenotype loss in age-related osteoarthritis. Front Pharmacol. 2024;15. DOI: 10.3389/fphar.2024.1476255. PMC11588467 39600372

[95] Martínez-Hernández A, Córdova EJ, Rosillo-Salazar O, García-Ortíz H, Contreras-Cubas C, Islas-Andrade S, Revilla-Monsalve C, Salas-Labadía C, Orozco L, Vanella L. Association of HMOX1 and NQO1 polymorphisms with metabolic syndrome components. PLOS ONE. 2015;10(5):e0123313. doi: 10.1371/journal.pone.0123313. 25933176 PMC4416764

[96] Zheng W, Tan TK, Paterson IC, Mutha NV, Siow CC, Tan SY, Old LA, Jakubovics NS, Choo SW, Biswas I. StreptoBase: an oral Streptococcus mitis group genomic resource and analysis platform. PLOS ONE. 2016;11(5):e0151908. doi: 10.1371/journal.pone.0151908. 27138013 PMC4854451

[97] Ruiz L, Margolles A, Sanchez B. Bile resistance mechanisms in Lactobacillus and Bifidobacterium. Front Microbiol. 2013;4:396. doi: 10.3389/fmicb.2013.00396. 24399996 PMC3872040

[98] Tanaka H, Hashiba H, Kok J, Mierau I. Bile salt hydrolase of Bifidobacterium longum —biochemical and genetic characterization. Appl Environ Microbiol. 2000;66(6):2502–2512. doi: 10.1128/AEM.66.6.2502-2512.2000. 10831430 PMC110569

[99] Alyssa G, Brenton P, Melinda AE. Bifidobacterium and the intestinal mucus layer. Microbiome Res Rep. 2023;2(4):36. doi: 10.20517/mrr.2023.37. 38045921 PMC10688832

[100] Wang C-M, Fernez MT, Woolston BM, Carrier RL. Native gastrointestinal mucus: critical features and techniques for studying interactions with drugs, drug carriers, and bacteria. Adv Drug Delivery Rev. 2023;200:114966. doi: 10.1016/j.addr.2023.114966. PMC11184232 37329985

[101] Glover JS, Ticer TD, Engevik MA. Characterizing the mucin-degrading capacity of the human gut microbiota. Sci Rep. 2022;12(1):8456. doi: 10.1038/s41598-022-11819-z. 35589783 PMC9120202

[102] Ruiz L, Gueimonde M, Couté Y, Salminen S, Sanchez J-C, de Los reyes-Gavilán CG, Margolles A. Evaluation of the ability of Bifidobacterium longum to metabolize human intestinal mucus. FEMS Microbiol Lett. 2011 [acccessed 2024 3 3];314(2):125–130. doi: 10.1111/j.1574-6968.2010.02159.x. 21105908

[103] Wu CM, Wheeler KM, Cárcamo-Oyarce G, Aoki K, McShane A, Datta SS, Mark Welch JL, Tiemeyer M, Griffen AL, Ribbeck K. Mucin glycans drive oral microbial community composition and function. NPJ Biofilms Microb. 2023;9(1):11. doi:10.1038/s41522-023-00378-4. PMC10036478 36959210

[104] Raimondi S, Musmeci E, Candeliere F, Amaretti A, Rossi M. Identification of mucin degraders of the human gut microbiota. Sci Rep. 2021;11(1):11094. doi: 10.1038/s41598-021-90553-4. 34045537 PMC8159939

[105] Candeliere F, Musmeci E, Sola L, Amaretti A, Raimondi S, Rossi M. Genomic and functional analysis of the mucinolytic species Clostridium celatum, Clostridium tertium, and Paraclostridium bifermentans. Front Microbiol. 2024;15. doi: 10.3389/fmicb.2024.1359726. PMC10952124 38511005

[106] Fiorucci S, Biagioli M, Zampella A, Distrutti E. Bile acids activated receptors regulate innate immunity. Front Immunol. 2018;9. doi: 10.3389/fimmu.2018.01853. PMC6099188 30150987

[107] van Rijn JM, van Hoesel M, de Heus C, van Vugt AM, Klumperman J, Nieuwenhuis ES, Houwen RJ, Middendorp S. DGAT2 partially compensates for lipid-induced ER stress in human DGAT1-deficient intestinal stem cells[S]. J Lipid Res. 2019;60(10):1787–1800. doi:10.1194/jlr.M094201. 31315900 PMC6795077

[108] Golden JM, Escobar OH, Nguyen MVL, Mallicote MU, Kavarian P, Frey MR, Gayer CP. Ursodeoxycholic acid protects against intestinal barrier breakdown by promoting enterocyte migration via EGFR- and COX-2-dependent mechanisms. Am J Physiol Gastrointestinal Liver Physiol. 2018;315(2):G259–g271. doi: 10.1152/ajpgi.00354.2017. PMC6139640 29672156

[109] Li T, Ding N, Guo H, Hua R, Lin Z, Tian H, Yu Y, Fan D, Yuan Z, Gonzalez FJ, et al. A gut microbiota-bile acid axis promotes intestinal homeostasis upon aspirin-mediated damage. Cell Host & Microbe. 2024 [acccessed 2025 Mar 13];32(2):191–208.e199. doi: 10.1016/j.chom.2023.12.015. 38237593 PMC10922796

[110] Luo J, Yang H, Song B-L. Mechanisms and regulation of cholesterol homeostasis. Nat Rev Mol Cell Biol. 2020;21(4):225–245. doi: 10.1038/s41580-019-0190-7. 31848472

[111] Abumrad NA, Davidson NO. Role of the gut in lipid homeostasis. Physiol Rev. 2012;92(3):1061–1085. doi:10.1152/physrev.00019.2011. 22811425 PMC3589762

[112] Jin D, Huang K, Xu M, Hua H, Ye F, Yan J, Zhang G, Wang Y. Deoxycholic acid induces gastric intestinal metaplasia by activating STAT3 signaling and disturbing gastric bile acids metabolism and microbiota. Gut Microbes. 2022;14(1):2120744. doi:10.1080/19490976.2022.2120744. 36067404 PMC9467587

[113] Sheng W, Ji G, Zhang L. The effect of lithocholic acid on the gut-liver axis. Front Pharmacol. 2022;13. doi: 10.3389/fphar.2022.910493. PMC9301130 35873546

[114] Gomes FMC, Geraldes CFG, Vaz WLC, Moreno MJ. Emulsification of cholesterol in bile Salt micelles: relevance for cholesterol absorption. Biophys J. 2010 [acccessed 2025 mar 13];98(3):80a. doi: 10.1016/j.bpj.2009.12.450.

[115] Mitchell MK, Ellermann M. Long chain fatty acids and virulence repression in intestinal bacterial pathogens. Front Cell Infect Microbiol. 2022;12. doi: 10.3389/fcimb.2022.928503. PMC9247172 35782143

[116] Ezzine C, Loison L, Montbrion N, Bôle-Feysot C, Déchelotte P, Coëffier M, Ribet D. Fatty acids produced by the gut microbiota dampen host inflammatory responses by modulating intestinal SUMOylation. Gut Microbes. 2022;14(1):2108280. doi:10.1080/19490976.2022.2108280. 35978476 PMC9466625

[117] Kastl AJ Jr, Terry NA, Wu GD, Albenberg LG. The structure and function of the human small intestinal microbiota: current understanding and future directions. Cellular Mol Gastroenterol Hepatol. 2020 [acccessed 2025 Mar 13];9(1):33–45. doi: 10.1016/j.jcmgh.2019.07.006. PMC6881639 31344510

[118] Hayashi H, Takahashi R, Nishi T, Sakamoto M, Benno Y. Molecular analysis of jejunal, ileal, caecal and recto-sigmoidal human colonic microbiota using 16S rRNA gene libraries and terminal restriction fragment length polymorphism. J Med Microbiol. 2005;54(11):1093–1101. doi:10.1099/jmm.0.45935-0. 16192442

[119] Kim HJ, Lee J, Choi J-H, Bahinski A, Ingber DE. Co-culture of living microbiome with microengineered human intestinal villi in a gut-on-a-chip microfluidic device. JOVE. 2016;114(114):e54344. doi: 10.3791/54344. PMC5091968 27684630

[120] Su X, Gao Y, Yang R. Gut microbiota derived bile acid metabolites maintain the homeostasis of gut and systemic immunity. Front Immunol. 2023;14. DOI: 10.3389/fimmu.2023.1127743. PMC10225537 37256134

