# Supplementary material for: Bacteria enable tolerance to bile salt exposure in an immune-competent human intestinal model
Source: Gut Microbes. 2025 Jun 21;17(1):2514144. doi: 10.1080/19490976.2025.2514144 (PMC12716047; doi:10.1080/19490976.2025.2514144)
Supplement: Bile salt research _supplemental materials.docx [file KGMI_A_2514144_SM8987.docx]

**Supplementary Data**


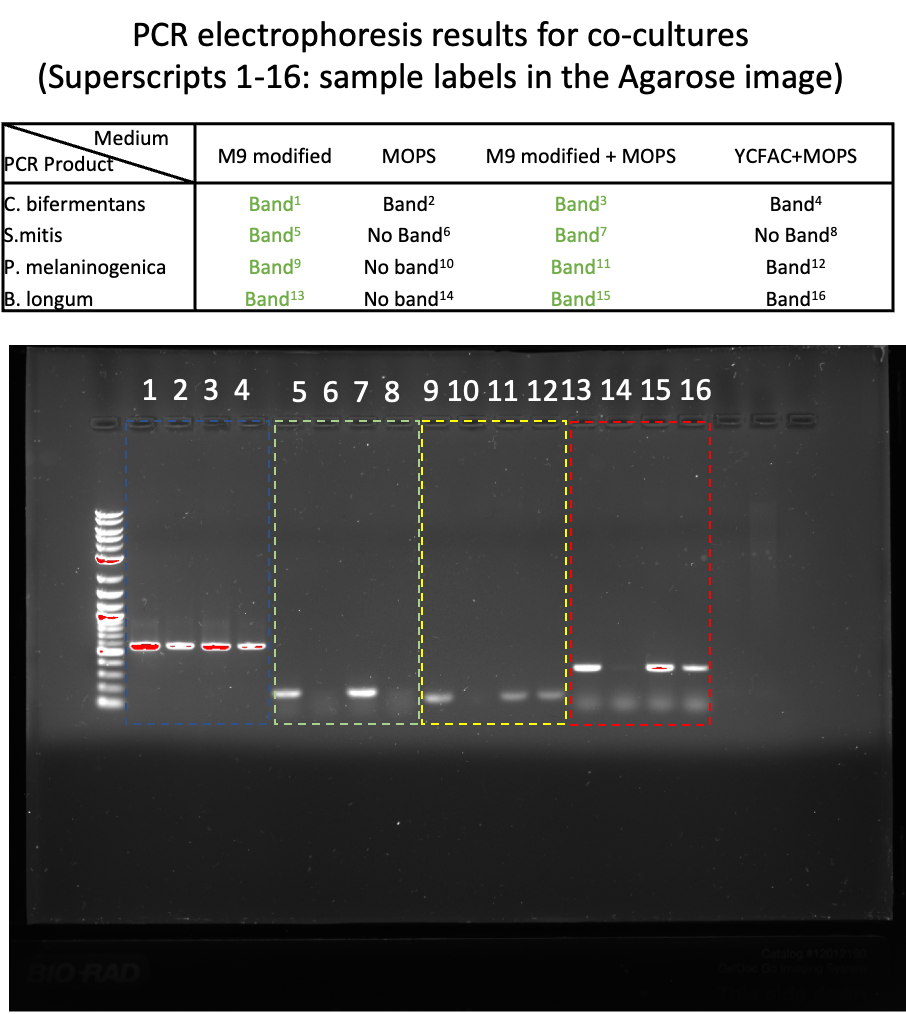


**Fig. S1. Chemically defined M9 modified medium supported the co-culture growth of all four bacterial strains.** Bacteria growth in four types of media is reflected in PCR electrophoresis. C. bifermentans, columns 1-4; S. mitis, columns 5-8; P. melaninogenica, columns 9-12; and B. longum, columns 13-16. M9 modified medium: columns 1, 5, 9, 13; EZ Rich Defined Medium (MOPS, Teknova, M2105): columns 2, 6. 10, 14; M9 modified/YCFAC (50%/50% v/v): columns 3, 7, 11, 15; YCFAC medium: columns 4, 8, 12, 16.

*
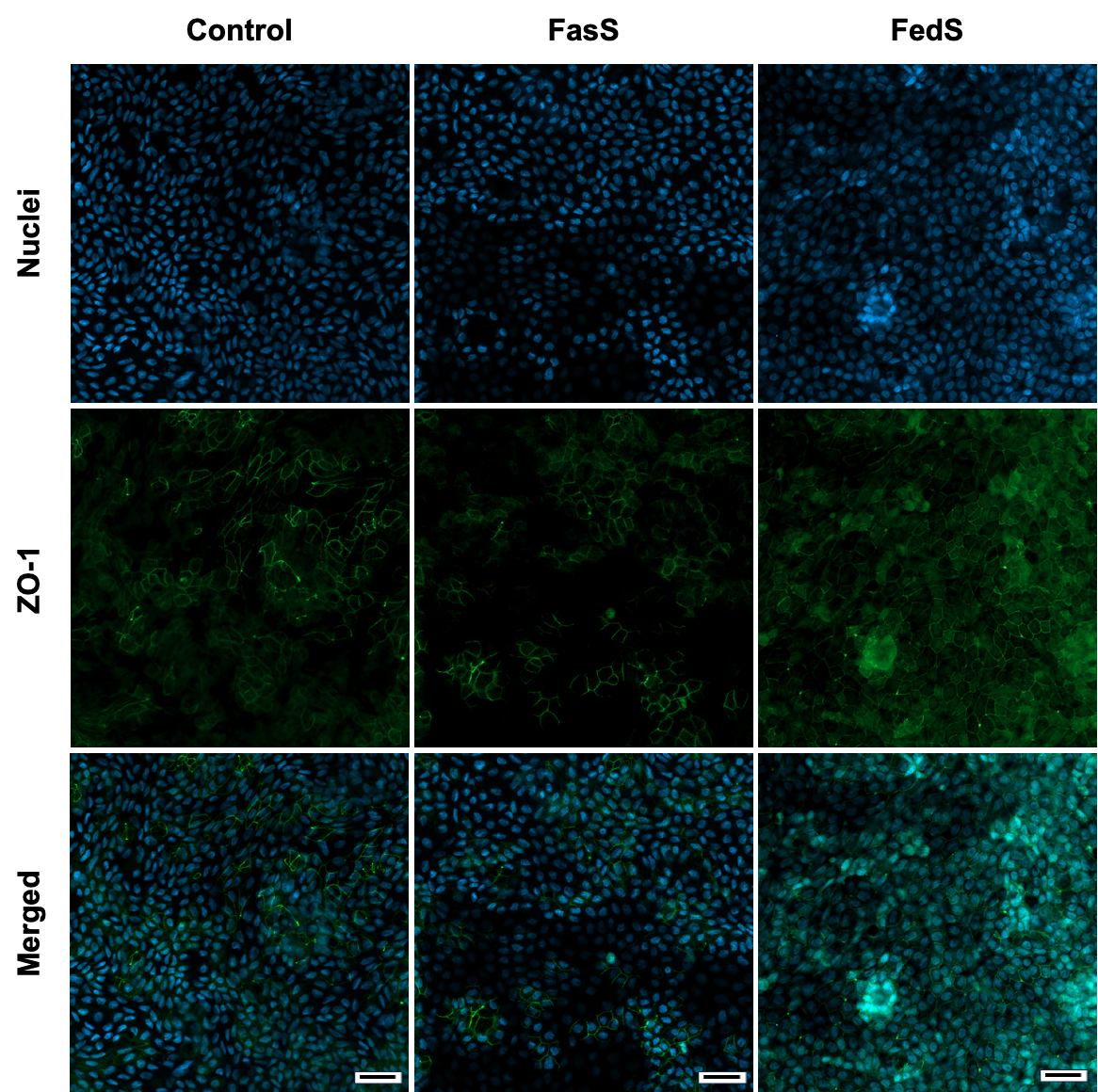
*

**Fig. S2. Fed State (FedS) exposure under normoxia impaired monolayer barrier function.** Monolayers exposed to FedS conditions exhibit disrupted ZO-1 tight junctions, with ZO-1 signal dispersing into the cytoplasm. Scale bar 50 μm. (**Control**: No BS/PC)

*
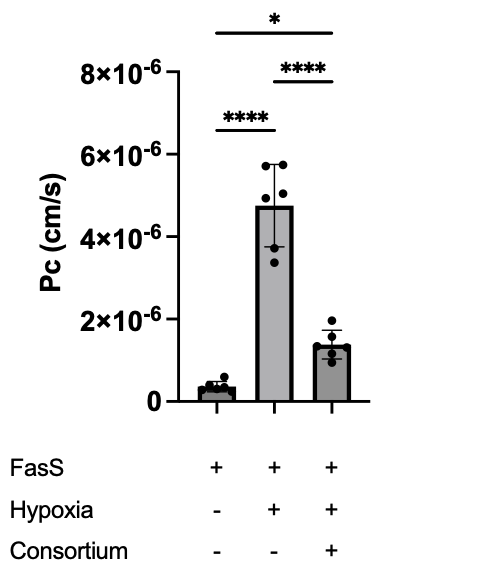
*

**Fig. S3. Hypoxic condition and consortium affect monolayer permeability after 24 hrs. Fasted State (FasS) exposure.** Significant differences from one-way ANOVA analysis: * p<0.05 **** p<0.0001.

*
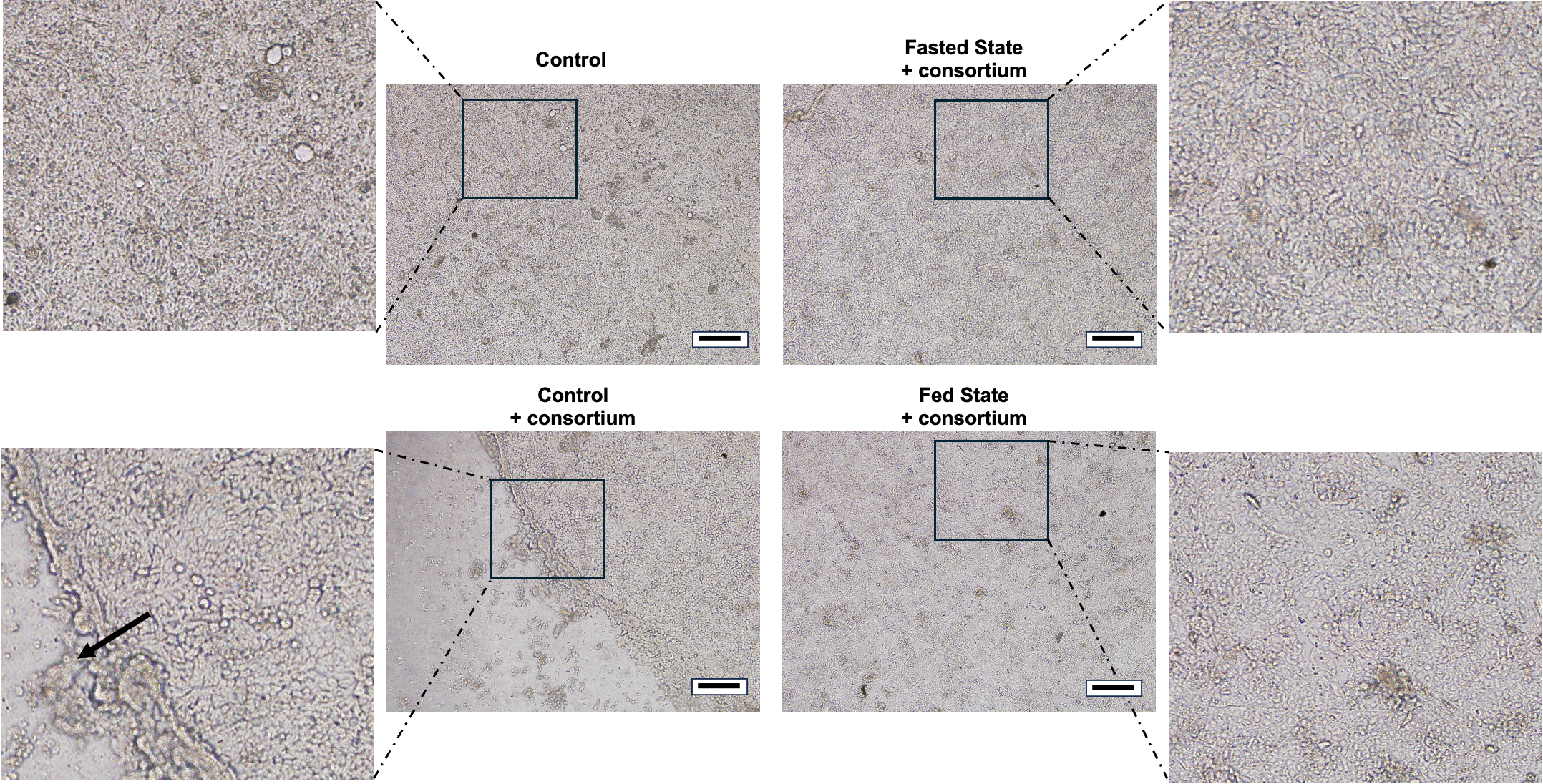
*

**Fig. S4. Damaged or transparent monolayer after consortium co-culture or BS/PC exposure.** The delamination and damage (arrow) of the monolayer caused by the bacterial co-culture were alleviated by the presence of bile, resulting in a relatively intact monolayer. In the Fed State + consortium condition, the boundaries between cells became less distinct, leading to a more transparent monolayer. Scale bar: 200 μm. (**Control**: No BS/PC)


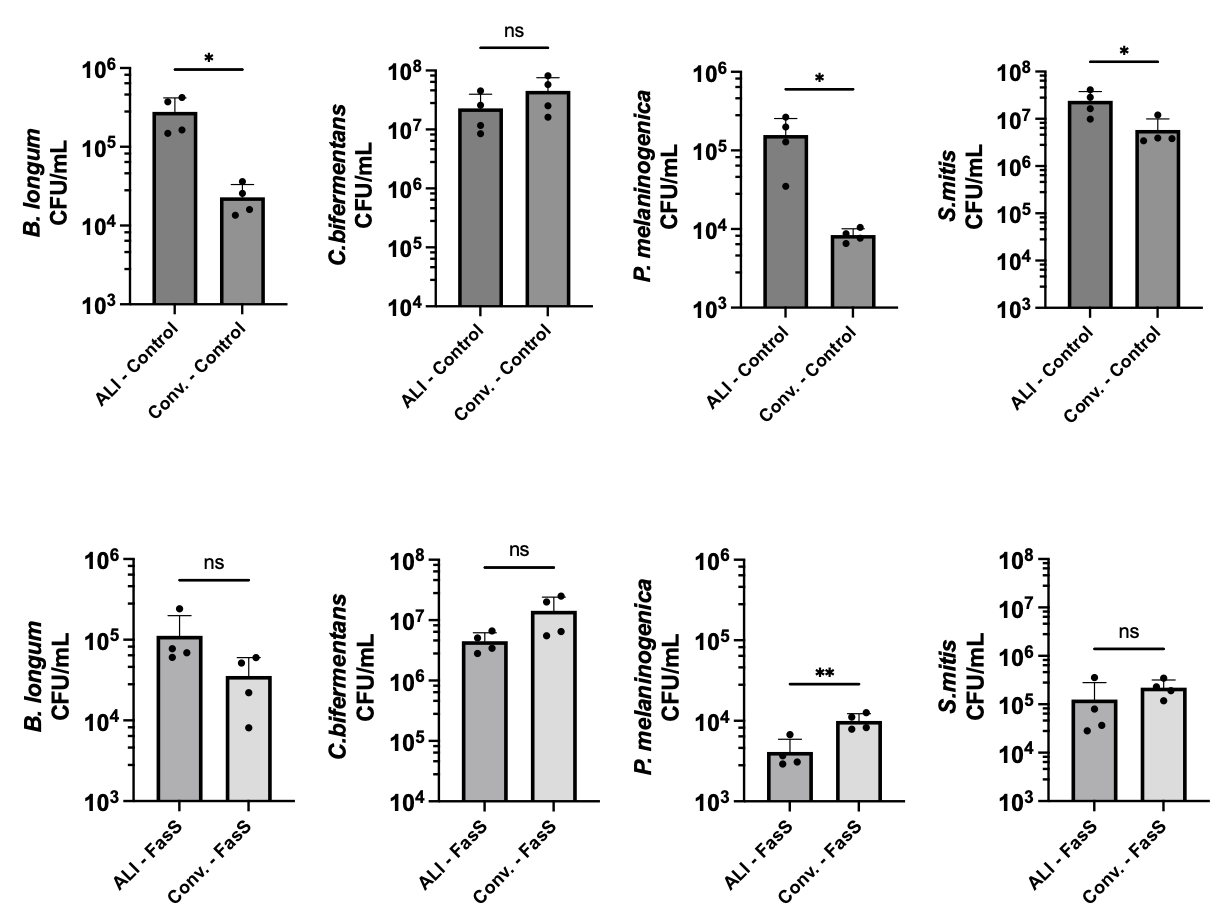


**Fig. S5. Strain densities varied between ALI and conventional cultures.** Replotted from Fig. 3B and 5C data. (**ALI - Control**: No BS/PC No OA, **Conv. – Control**: No BS/PC) Significant differences from unpaired t-test analysis: * p<0.05, ** p<0.01.


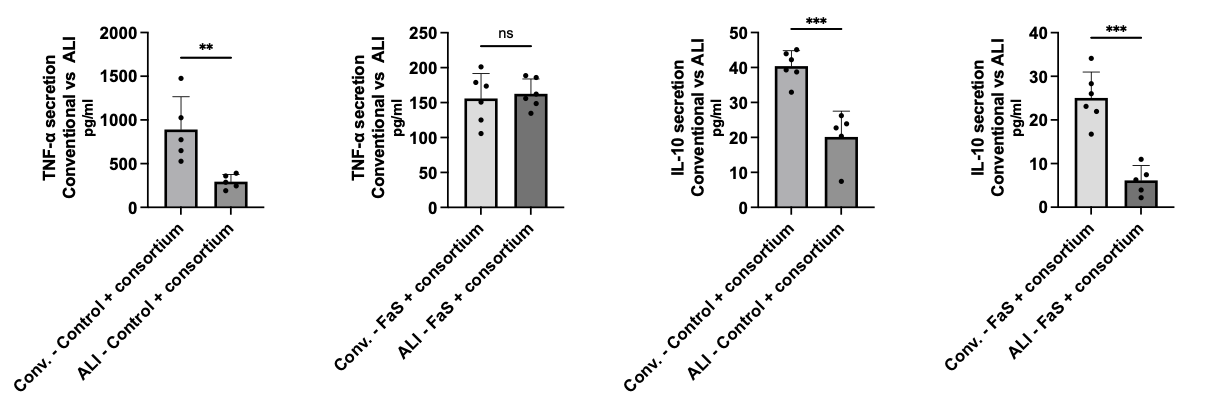


**Fig. S6. ALI culture resulted in lower inflammatory responses compared to conventional culture.** Replotted from Fig. 3E and 6D data. (**ALI - Control**: No BS/PC No OA, **Conv. – Control**: No BS/PC) Significant differences from unpaired t-test analysis: * p<0.05, ** p<0.01, *** p<0.001, **** p<0.0001.

**Table S1. DM formulation for conventional culture**

*
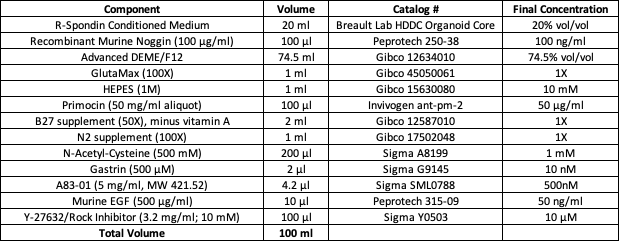
*

**Table S2. Chemical defined M9 medium formulation**


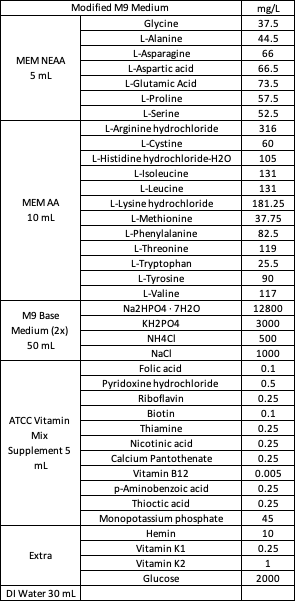


**MEM NEAA**: MEM Non-essential Amino Acids Solution (Gibco, 11140050)

**MEM AA**: MEM Amino Acids Solution (Gibco, 11130051)

**ATCC Vitamin Mix** (ATCC, MD-VS)

**Table S3. Primer sequences for bacterial consortium**

| Microbe | Primer | Sequence 5'-3' | Reference |
| --- | --- | --- | --- |
| *B. longum KLE 2100* | Forward | CGGTCGTAGAGATACGGCTT | [1] |
|  | Reverse | ATCCGAACTGAGACCGGTT |  |
| *C. bifermentans ATCC 638* | Forward | CAAGTCGAGCGATCTCT | [2] |
|  | Reverse | CCTGCACTCAAGTTCTCT |  |
| *S. mitis NCIMB 13770* | Forward | AGGATAAGGAACTGCACATTGGTC | [3] |
|  | Reverse | TGCATTACTTGGTGATCTCTCACC |  |
| *P. melaninogenica ATCC 25845* | Forward | GTGGGATAACCTGCCGAAAG | [4] |
|  | Reverse | CCCATCCATTACCGATAAATCTTTA |  |

**Table S4. Gene expression assay information**

| **TaqMan gene expression assays** | **Assay ID** |
| --- | --- |
| GAPDH | Hs04420697_g1 |
| PDK1 | Hs01561847_m1 |
| YAP1 | Hs00902712_g1 |
| Nrf2 | Hs00975961_g1 |
| DGAT1 | Hs01020362_g1 |
| DGAT2 | Hs02786624_g1 |

**References:**

(1) Kwon, H.-S.; Yang, E.-H.; Lee, S.-H.; Yeon, S.-W.; Kang, B.-H.; Kim, T.-Y. Rapid identification of potentially probiotic Bifidobacterium species by multiplex PCR using species-specific primers based on the region extending from 16S rRNA through 23S rRNA. *FEMS Microbiology Letters* **2005**, *250* (1), 55-62. DOI: 10.1016/j.femsle.2005.06.041 (acccessed 12/21/2024).

(2) Kikuchi, E.; Miyamoto, Y.; Narushima, S.; Itoh, K. Design of Species-Specific Primers to Identify 13 Species of Clostridium Harbored in Human Intestinal Tracts. *Microbiology and Immunology* **2002**, *46* (5), 353-358. DOI: <https://doi.org/10.1111/j.1348-0421.2002.tb02706.x>.

(3) Hoshino, T.; Izumi, T.; Ooshima, T.; Fujiwara, T. Method for rapid identification of oral streptococci by PCR using 16S-23S ribosomal RNA intergenic spacer gene. *Pediatric Dental Journal* **2005**, *15* (2), 185-190. DOI: <https://doi.org/10.1016/S0917-2394(05)70051-3>.

(4) De Martin, A.; Lütge, M.; Stanossek, Y.; Engetschwiler, C.; Cupovic, J.; Brown, K.; Demmer, I.; Broglie, M. A.; Geuking, M. B.; Jochum, W.; et al. Distinct microbial communities colonize tonsillar squamous cell carcinoma. *OncoImmunology* **2021**, *10* (1), 1945202. DOI: 10.1080/2162402X.2021.1945202.
